# The Structural Architecture of an Infectious Mammalian Prion Using Electron Cryomicroscopy

**DOI:** 10.1371/journal.ppat.1005835

**Published:** 2016-09-08

**Authors:** Ester Vázquez-Fernández, Matthijn R. Vos, Pavel Afanasyev, Lino Cebey, Alejandro M. Sevillano, Enric Vidal, Isaac Rosa, Ludovic Renault, Adriana Ramos, Peter J. Peters, José Jesús Fernández, Marin van Heel, Howard S. Young, Jesús R. Requena, Holger Wille

**Affiliations:** 1 Department of Biochemistry, University of Alberta, Edmonton, Alberta, Canada; 2 Centre for Prions and Protein Folding Diseases, University of Alberta, Edmonton, Alberta, Canada; 3 FEI Company, Nanoport Europe, Eindhoven, The Netherlands; 4 Institute of Biology Leiden, NeCEN, Leiden, The Netherlands; 5 The Maastricht Multimodal Molecular Imaging Institute, Maastricht University, Maastricht, The Netherlands; 6 CIMUS Biomedical Research Institute University of Santiago de Compostela-IDIS, Santiago de Compostela, Spain; 7 IRTA, Centre de Recerca en Sanitat Animal (CReSA, IRTA-UAB), Campus de la Universitat Autònoma de Barcelona, Bellaterra, Catalonia, Spain; 8 Centro Nacional de Biotecnologia - CSIC, Campus Universidad Autónoma, Madrid, Spain; 9 Brazilian Nanotechnology National Laboratory - LNNano, CNPEM, Campinas, São Paulo, Brazil; 10 Faculty of Natural Science, Imperial College London, London, United Kingdom; Dartmouth Medical School, UNITED STATES

## Abstract

The structure of the infectious prion protein (PrP^Sc^), which is responsible for Creutzfeldt-Jakob disease in humans and bovine spongiform encephalopathy, has escaped all attempts at elucidation due to its insolubility and propensity to aggregate. PrP^Sc^ replicates by converting the non-infectious, cellular prion protein (PrP^C^) into the misfolded, infectious conformer through an unknown mechanism. PrP^Sc^ and its N-terminally truncated variant, PrP 27–30, aggregate into amorphous aggregates, 2D crystals, and amyloid fibrils. The structure of these infectious conformers is essential to understanding prion replication and the development of structure-based therapeutic interventions. Here we used the repetitive organization inherent to GPI-anchorless PrP 27–30 amyloid fibrils to analyze their structure via electron cryomicroscopy. Fourier-transform analyses of averaged fibril segments indicate a repeating unit of 19.1 Å. 3D reconstructions of these fibrils revealed two distinct protofilaments, and, together with a molecular volume of 18,990 Å^3^, predicted the height of each PrP 27–30 molecule as ~17.7 Å. Together, the data indicate a four-rung β-solenoid structure as a key feature for the architecture of infectious mammalian prions. Furthermore, they allow to formulate a molecular mechanism for the replication of prions. Knowledge of the prion structure will provide important insights into the self-propagation mechanisms of protein misfolding.

## Author Summary

The structure of the infectious prion (PrP^Sc^), which is responsible for Creutzfeldt-Jakob disease in humans and bovine spongiform encephalopathy, has escaped all attempts at elucidation due to its propensity to aggregate. Here, we use the repetitive organization inherent in amyloid fibrils to analyze the structure of GPI-anchorless PrP 27–30 via electron cryomicroscopy. Fourier-transform analysis of averaged fibril segments indicates a repeating unit of 19.1 Å. In agreement with this observation, 3D reconstructions reveal that each fibril contains two distinct protofilaments and that the height of each PrP 27–30 molecule in these fibrils is ~17.7 Å. Together the data indicate a four-rung β-solenoid structure as a key feature for the architecture of infectious mammalian prions. The data conflict with all previous models for the structure of PrP^Sc^ and allow the formulation of a molecular mechanism for the replication of prions.

## Introduction

Little is known about the structure of the infectious prion protein, the infectious agent causing prion diseases such as sheep and goat scrapie, bovine spongiform encephalopathy or “mad cow disease”, chronic wasting disease in cervids (deer, elk, moose, and reindeer), and Creutzfeldt-Jakob disease in humans. The structure of these infectious conformers is essential to understanding prion replication and the development of structure-based therapeutic interventions. The non-infectious, cellular prion protein (PrP^C^), which has its highest expression levels in neurons, is misfolded through a posttranslational process into an altered, infectious conformer termed PrP^Sc^ or prion [1]. The structure of recombinant PrP, which approximates the structure of PrP^C^, has been solved repeatedly by NMR spectroscopy [2] and X-ray crystallography [3]. PrP^C^ consists of an unfolded N-terminal domain and a largely α-helical C-terminal domain, which contains three α-helices and a short, two-stranded ß-sheet [2,3]. In contrast, PrP^Sc^ has been found by a variety of methods to contain predominantly ß-sheet structure [4].

PrP^Sc^ and its N-terminally truncated variant, PrP 27–30, are generally insoluble and prone to aggregation into an assortment of quaternary structures, such as amorphous aggregates, 2D crystals, and amyloid fibrils. None of these aggregation products are amenable to conventional structural analysis techniques such as X-ray crystallography or solution NMR spectroscopy. To overcome the paucity of experimental data on the structure of the infectious prion, molecular modeling has been used by a variety of researchers to predict its structure. Little agreement exists among the published molecular models regarding the nature of the infectious conformer and a large range of different folds have been put forward [5], with the most recent entry proposing a parallel in-register ß-sheeted structure [6]. While none of the published models satisfy all experimental restraints [5], a ß-helical architecture has been suggested as a likely candidate for the structure of the infectious prion [7,8].

To investigate the structure of the infectious prion, we used electron cryomicroscopy (cryo-EM) to record and analyze the structure of brain-derived, murine prion protein amyloid. Brain-derived PrP^Sc^ has a high level of molecular heterogeneity due to its GPI-anchor and N-linked carbohydrates, which increases the difficulty to analyze its structure considerably. On the other hand, brain-derived PrP^Sc^, as the disease-relevant conformer, can provide insights into the infectious state, which the more well-behaved, recombinantly-derived PrP amyloid, cannot [9]. By using transgenic mice expressing a GPI-anchorless form of the prion protein, which is also substantially underglycosylated [10], a more homogeneous version of the prion protein could be analyzed. GPI-anchorless PrP^Sc^ is deposited predominantly as large fibrillar amyloid plaques, which allows for a milder purification procedure compared to traditional purification approaches [11]. In prion-infected mice expressing GPI-anchorless PrP, PrP^Sc^ retains its full infectivity, while the neuropathology is similar to a cerebral amyloid angiopathy (CAA) as seen in Alzheimer’s disease [12] and hereditary prion protein amyloidosis [13,14]. This difference in apparent neuropathology is not surprising given that infectivity and toxicity of PrP^Sc^ are not unequivocally linked [15]. After all, GPI-anchorless PrP^Sc^ cannot attach to the cell membrane, and therefore exhibits a different cellular and tissue distribution. Nonetheless, the homogeneous preparation of GPI-anchorless, infectious prions facilitated novel insights into the structure of PrP^Sc^ by cryo-EM.

## Results

### Characterization and preparation of GPI-anchorless prion fibrils

**Fig 1:**
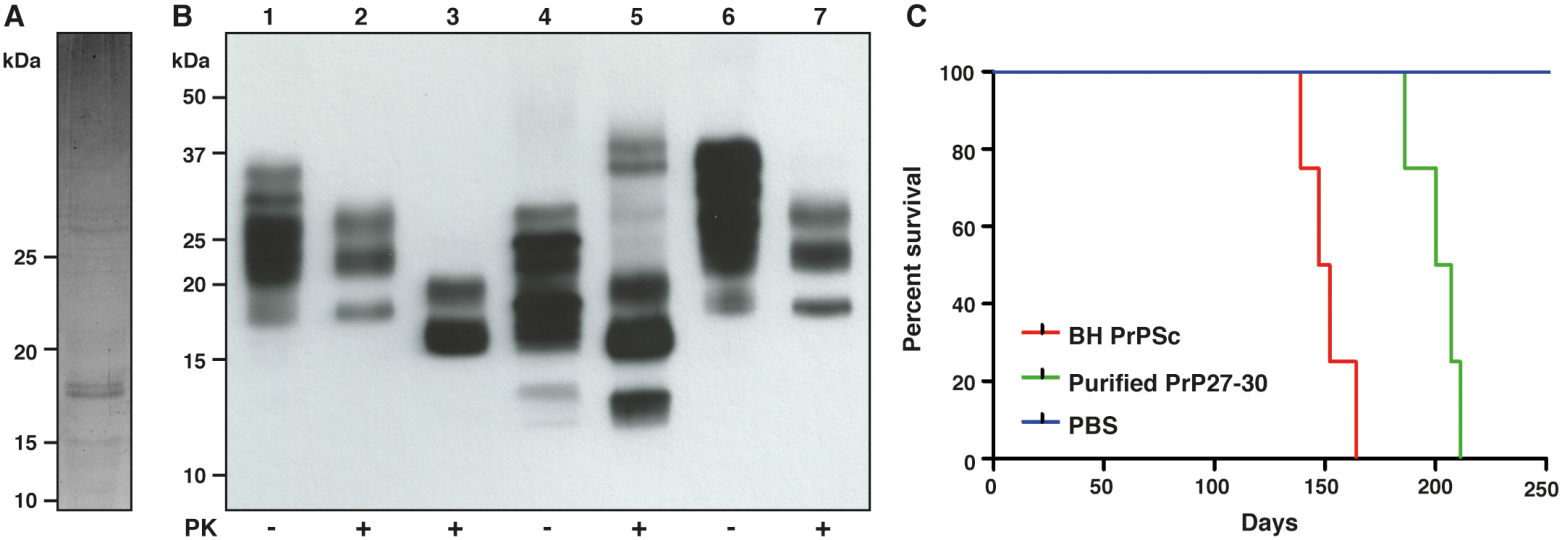
Characterization of GPI-anchorless prions. (**A**) SYPRO Ruby-stained 15% SDS-PAGE of purified GPI-anchorless PrP 27–30. The molecular mass of the purified peptide (residues 89–232) is 17,148 Da. The doublet seen at ~17 kDa reflects the presence of PK-resistant fragments of slightly different sizes (“ragged ends” [18]). Faint bands of ~34 kDa are characteristic dimers, the result of an incomplete dissociation of PrP 27–30 fibrils. Minor impurities correspond to traces of ferritin, tubulin and collagen [23], which are clearly recognizable in electron micrographs. (**B**) Western blot of: Lanes 1–2, RML-infected WT mouse brain homogenate (BH), ± PK digestion. WT PrP presents as three characteristic bands of di-, mono-, and unglycosylated protein, respectively. Lanes 3–4, RML-infected GPI-anchorless PrP transgenic mouse BH, ± PK digestion; lane 5, purified GPI-anchorless PrP 27–30, used for cryo-EM studies; the 10–15 kDa band corresponds to a minor population of N-terminally truncated PK-resistant fragments described by Vázquez-Fernández et al. [18]; lanes 6–7, WT mouse BH inoculated with purified GPI-anchorless PrP 27–30, ± PK digestion. (**C**) Kaplan-Meier survival analysis of: WT mice infected with GPI-anchorless PrP^Sc^ BH (red), survival time 153 ± 10 days (standard deviation); WT mice infected with purified GPI-anchorless PrP 27–30 (green), survival time 203 ± 9 days (standard deviation); WT mice inoculated with PBS as negative control (blue) (n = 6, *P* < 0.05, Gehan-Breslow-Wilcoxon test).

**Fig 2:**
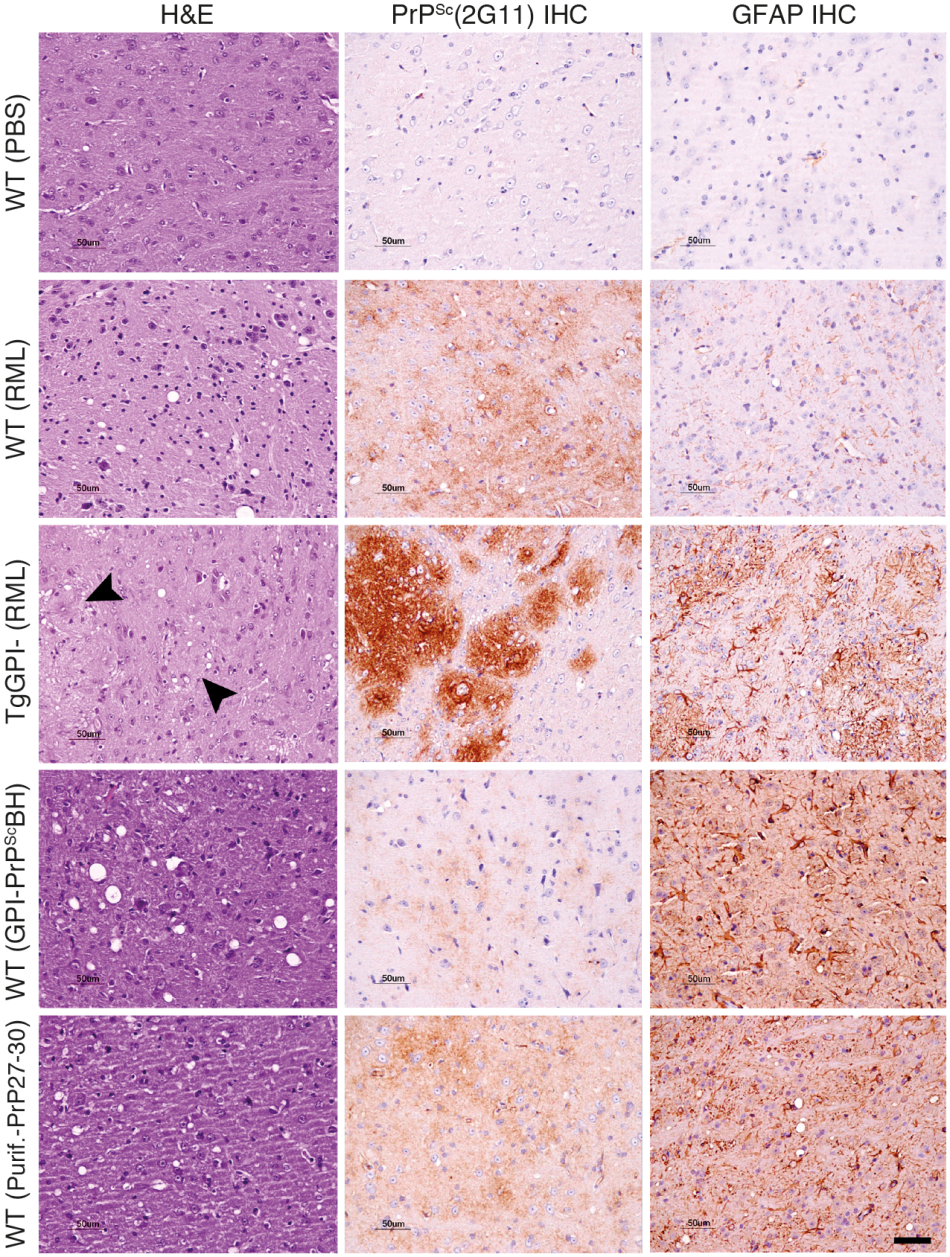
Histopathological and immunohistochemical analyses. Brain tissue sections of the thalamus: one section was stained with hematoxylin and eosin (H&E, left), two others were analyzed via immunohistochemistry (IHC) using the anti-PrP antibody 2G11 (middle) and an antibody against the glial fibrillary acidic protein (GFAP) to reveal astroglial activation (right). Control WT mice inoculated with PBS (first row); WT mice infected with RML prions (second row); and transgenic mice expressing GPI-anchorless PrP infected with RML prions (third row). Black arrowheads indicate the presence of hyaline deposits, which stain PrP angiocentric plaques. WT mice infected with GPI-anchorless PrP^Sc^ BH (fourth row); and WT mice infected with purified GPI-anchorless PrP 27–30 (fifth row).

GPI-anchorless PrP 27–30, which, for consistency, is named according to the molecular weight of GPI-anchored PrP 27–30, was purified from the brains of transgenic mice expressing GPI-anchorless PrP that were infected with prions of the Rocky Mountain Laboratory (RML) strain [10,16]. The purification procedure took advantage of both the *in vivo* formed amyloid fibrils of GPI-anchorless PrP^Sc^ and their partial resistance against Proteinase K (PK) digestion. Limited proteolysis of GPI-anchorless PrP^Sc^, similar to the digestion of GPI-anchored PrP^Sc^, removes just the first ~66 N-terminal residues, leaving a ~17 kDa PK-resistant, GPI-anchorless, unglycosylated fragment, and a very modest amount of monoglycosylated protein [10,17,18] (Fig 1A and 1B). To verify that the purified GPI-anchorless PrP 27–30 preparations were still infectious, as reported by others, we inoculated a cohort of wild-type mice with the purified prions. All animals developed the typical neurological signs of RML-prion disease. Furthermore, biochemical and histopathological analyses demonstrated that the brains of infected mice showed all hallmarks of pathogenic prion disease, for example partial PK-resistance, presence of vacuolation, gliosis, and PrP^Sc^ deposition in the brain tissue (Figs 1B and 2). The observed, moderately-extended incubation period compared to animals inoculated with untreated brain homogenate (Fig 1C) is compatible with the use of PK treatment, which is known to degrade PrP^C^ and PK-sensitive forms of PrP^Sc^, thereby reducing the apparent prion titer [19,20]. In addition, the highly aggregated nature of the purified prions is also known to lower the effective prion titer [21,22].

### Cryo-EM analysis of GPI-anchorless prion fibrils

**Fig 3:**
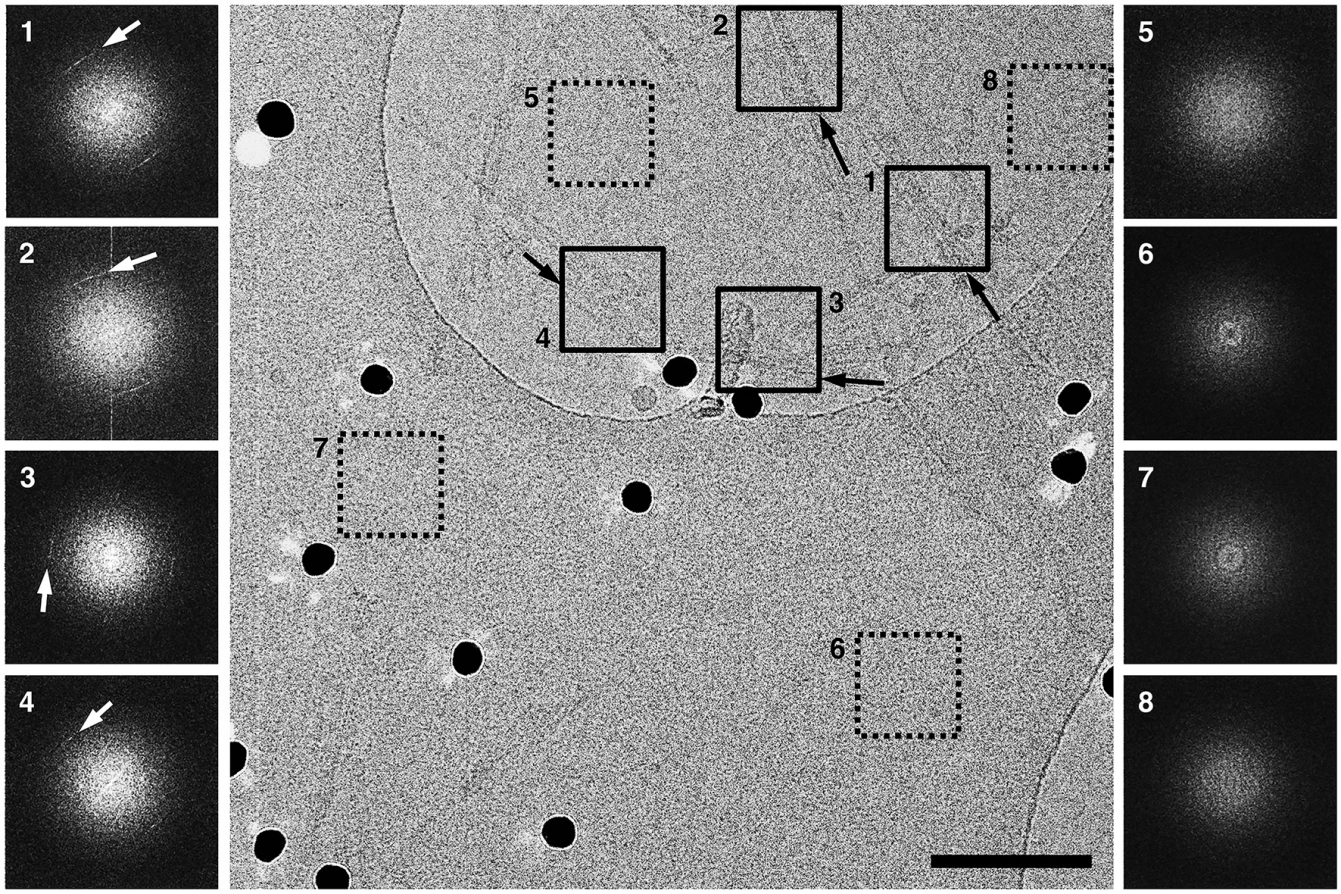
Raw cryo-EM images of GPI-anchorless prion fibrils show their basic features and 4.8 Å cross-β signals. A high-resolution cryo-EM image showing individual GPI-anchorless PrP 27–30 fibrils or small bundles of fibrils (solid boxes). Fourier-transform (FT) analyses from the corresponding boxes show 4.8 Å cross-ß signals that originate from the ß-strand stacking along the fibril axis and which follow the orientation of the fibrils (black and white arrows). FT analyses of areas of ice or carbon film, that were devoid of amyloid fibrils (dotted boxes), and which did not show any noticeable signals at 4.8 Å. Scale bar, 100 nm.

**Fig 4:**
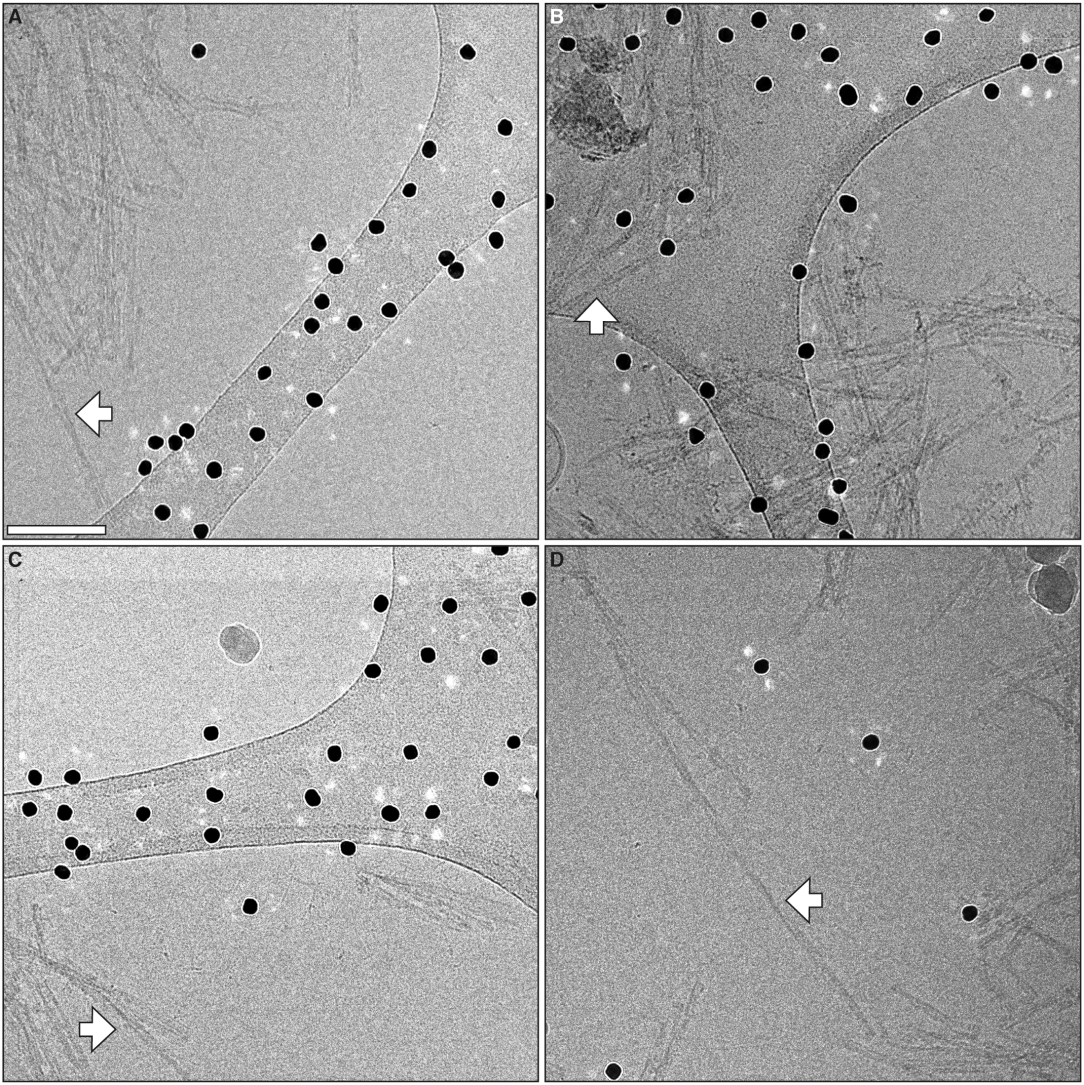
Additional cryo-EM micrographs of GPI-anchorless prion fibrils. Electron micrographs showing representative GPI-anchorless prion fibrils, including four isolated fibrils that were subsequently analyzed by image processing (white arrows). The labels (**A** to **D**) correspond to the lettering in the 3D fibril reconstruction figures (*vide infra*). Black dots originate from fiducial gold that was added for tomographic studies. Scale bar, 100 nm.

The cryo-EM images from GPI-anchorless PrP 27–30 fibrils were examined in detail. Images showed fibrils ~10 nm wide, composed by two intertwined, twisting protofilaments, with a space between them (Figs 3, 4 and S1). A clearer view of the fibrils was obtained in 3D tomograms (S1 Video and S2 Fig), which allowed a more facile visualization of individual fibrils. Separate fibrils were found to display either a left- or right-handed twist, or to be essentially straight. This apparent variability with respect to the fibril helicity of GPI-anchorless fibrils had been observed before in negatively stained sample preparations [17]. Reconstructed tomograms allowed measurement of fibril widths, which were confirmed to be 9.55 ± 1.15 nm (standard deviation; n = 261) (S1 Fig). The limited dispersion of the values is similar to that of other amyloids [24].

The raw images contain high-resolution information, as surmised from Fourier-transform analyses routinely showing a 4.8 Å cross-β signal for individual fibrils along their axis (Fig 3) that can be readily interpreted as originating from β-strands running perpendicular to the fibril axis. It is notable that the orientation of the cross-β signal is strictly dependent on the orientation of the individual amyloid fibrils, which proves its origin (Fig 3, **black and white arrows**). Furthermore, Fourier-transform analyses of nearby empty areas of ice or carbon film never showed a 4.8 Å signal (Fig 3, **dotted boxes**), indicating that the 4.8 Å signal is indeed a cross-β signal similar to what was seen in X-ray fiber diffraction from PrP 27–30 [25]. In fact, to our knowledge, this is the first time that electron cryomicroscopy and the use of a second generation direct electron detector (Falcon II) has allowed to detect the 4.8 Å cross-β signal in Fourier-transform analyses of otherwise unprocessed cryo low-dose electron micrographs of individual amyloid fibrils or small bundles of fibrils.

Encouraged by this, we set out to extract as much additional structural information from the images as possible. In particular, we aimed at identifying the individual PrP subunits stacked in each protofilament. Unfortunately, fibrils were highly aggregated and did not display a constant helical periodicity. This impeded the use of the iterative helical real-space reconstruction (IHRSR) algorithm, which has frequently been used to process electron micrographs of amyloid fibrils to obtain high-resolution structural information [26]. Instead, we applied two single particle approaches to analyze a large number of short fibril segments from high magnification electron micrographs.

### Single particle analysis

**Fig 5:**
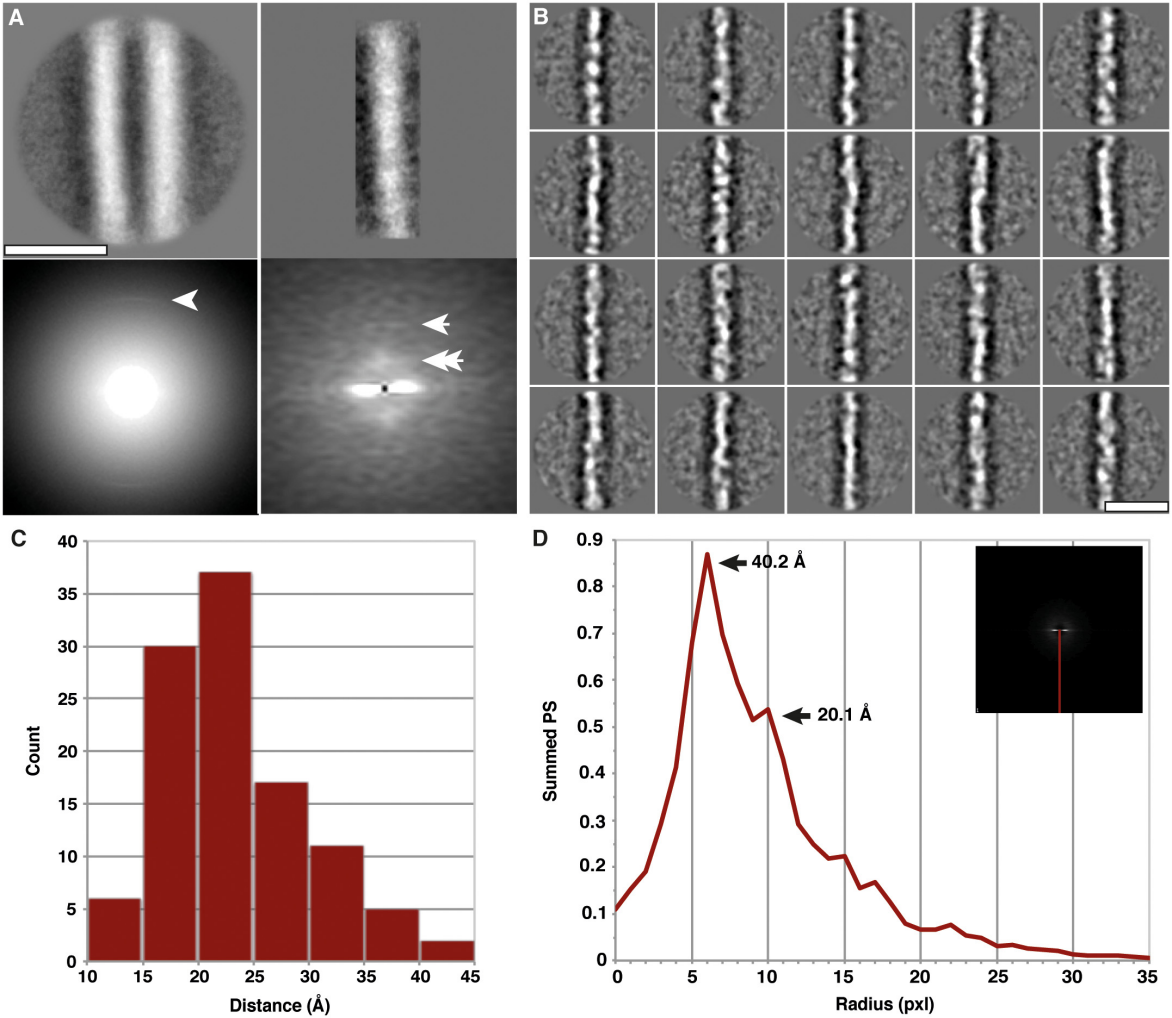
Single particle averaging of GPI-anchorless PrP 27–30 fibril images. (**A**) Average from 1072 fibril segments (top left) and the logarithm of their summed power spectrum (bottom left). The arrowhead indicates the characteristic 4.8 Å cross-β signal. Representative 2D class average of 100 single protofilaments (top right) and their averaged Fourier-transform (bottom right). Single and double arrows are pointing to intensities at 19.1 Å (single pixel) and ~40 Å (38.3 Å and 44.6 Å pixels), respectively. (**B**) Gallery of 2D class averages obtained from reference-free analysis of individual protofilaments. Box size is 150 by 150 pixels, equivalent to 20.1 by 20.1 nm. (**C**) Histogram of manually determined sizes of densities along the protofilament from the class averages in (B). The majority of densities fall into the classes between 15 and 25 Å. (**D**) The average over all amplitude spectra of all class averages. A plot of the meridian of the Fourier-transform (red line) reveals a broad peak around 40.2 Å and a smaller peak at 20.1 Å (arrows). The Nyquist frequency (2.68 Å) corresponds with the 75 pixel outer border of the spectra. Scale bars, 10 nm.

In the first single particle analysis approach, cryo-EM images were selected that presented clear Thon rings to enable correction of the contrast transfer function (CTF). In total, we extracted 1305 non-overlapping isolated fibril segments that were then aligned, classified, and averaged (S3 Fig). An averaged power spectrum of 1072 aligned segments showed a 4.8 Å intensity (Figs 5A and S4), characteristic of the cross-β structure of amyloid fibrils, also detected in many raw images (*vide supra*). The arc-shape of the 4.8 Å signal was indicative of an imperfect alignment of the fibrils. To overcome this problem, we analyzed each of the protofilaments that make up the fibril individually, which revealed 19.1 Å and ~40 Å signals upon Fourier-transform analysis (Figs 5A, S4 and S5). These spacings correspond to 4 and 8 multiples of 4.8 Å β-strands, and indicate the existence of a structural subunit with a height of 4 β-strands, that associates vertically with another subunit to form a higher order dimeric structure. Another feature, the absence of a strong ~10 Å signal on the equator of the Fourier-transforms (Figs 5A and S5), is generally interpreted to indicate the presence of β-helical and β-solenoidal structures [25,27]. The ~10 Å signal is commonly seen with stacked β-sheet structures such as Aβ(1–40) amyloid [28], and given the fact that our electron micrographs readily display the 4.8 Å cross-β signal in Fourier-transform analyses of individual fibrils (Fig 3), the ~10 Å signal could be expected if a stacked β-structure were present. Therefore, the most parsimonious explanation of this spacing hierarchy is the presence of a 4-rung β-solenoid as the basic subunit along the protofilament axis.

In an alternative reference-free single particle approach, we selected 2725 fibril segments from individual protofilaments, and performed eigenvector data compression and unsupervised automatic classification (Fig 5B). This analysis was performed without alignments and thereby avoiding reference bias (see materials and method for details and S6 Fig). The average of the amplitude spectra of all 20 classes (Fig 5B) revealed peaks at ~20 Å and ~40 Å (Fig 5D), in good agreement with the first approach. Furthermore, individual class averages (Fig 5B) showed distinct densities along the fibril axis with an average height of ~20 Å (Fig 5C), corroborating the other measurements.

### 3D fibril reconstructions

Considering all this, we reasoned that the volumetric information provided by individual fibrils should provide an independent assessment of the conclusion that GPI-anchorless subunits are ~20 Å "tall". We picked isolated fibrils with images clearly showing a crossover point and covering at least one half turn (180°) without overlap to other fibrils. Given the already mentioned extensive clumping of the fibrils, which showed a high degree of lateral aggregation and, most of the times, overlapped with neighboring fibrils, only a few fibrils meeting these visual requirements were selected (Figs 4 and S1). Based on their morphology and dimensions, these fibrils are representative of the majority of specimens seen in the micrographs, and no bias, other than the mentioned selection requirements, took place to choose them.

We then generated 3D reconstructions of these fibrils, taking advantage of the fact that the 2D image of a helical object contains rotated projections of its 3D surface. Thus, we segmented the isolated fibrils into overlapping boxes along the helical axis and analyzed the fibril segments as single objects. Each box is a different view of identically the same fibril rotated around and translated along the helical axis [29]. By measuring the helical repeat distance of the fibril (images shown in Fig 4) we could estimate the angular orientation of each box in the set (S7 and S8 Figs).

**Fig 6:**
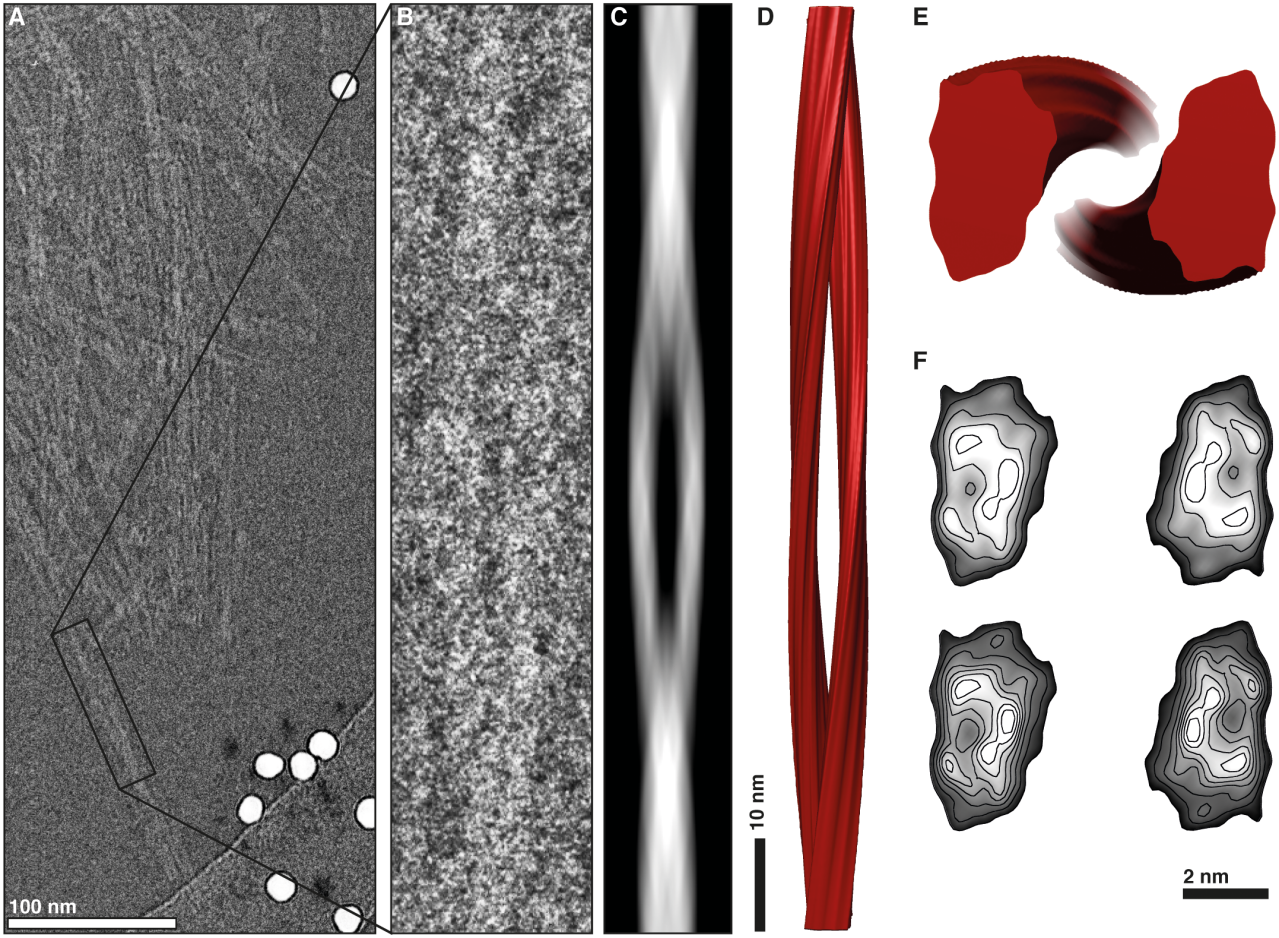
3D reconstruction of a GPI-anchorless prion fibril. (**A**) Section of an electron micrograph showing GPI-anchorless prion fibrils. The single isolated and twisted fibril used for the 3D reconstruction is enclosed by a black box. (**B**) Close-up view of the prion fibril. (**C**) Re-projected image of the 3D fibril map. (**D**) Reconstruction of the GPI-anchorless prion fibril. (**E**) Cross-section of the reconstructed fibril. (**F**) Contoured density maps of the cross-sections. Lines are contoured at increasing levels of 0.25 σ and 0.125 σ (top and bottom, respectively).

**Fig 7:**
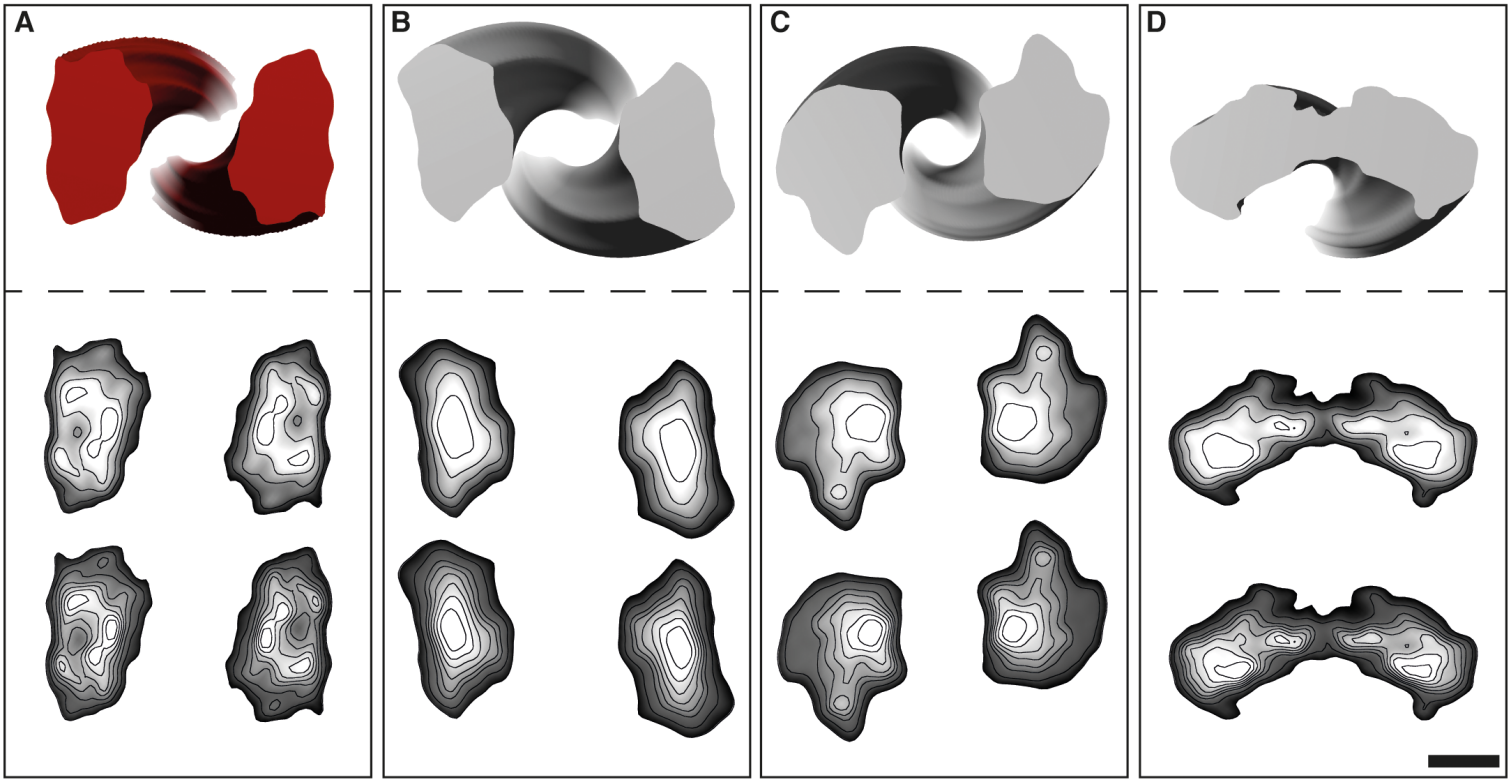
Cross-sections and density contour plots. Cross-sections from the four single GPI-anchorless prion fibrils that were analyzed (top). Contoured density lines from the cross-sections of the 3D maps in the yz plane (bottom). Density maps are contoured at increasing levels of 0.25 σ and 0.125 σ (top and bottom, respectively). Labels from (**A**–**D**) are in concordance with Figs 4 and S9. Scale bar, 2 nm.

The 3D reconstruction of one of these individual GPI-anchorless PrP 27–30 fibrils, with a maximum width of 9.1 nm and a crossover distance of 95 nm, showed two approximately 50 x 29 Å oval-shaped protofilaments (Fig 6). Fibril reconstruction statistics are listed in S1 Table. The density profile of the cross-section suggested several densities distributed within the core of the protofilament (Fig 6F), compatible with a ß-helical or ß-solenoidal fold and similar to what has been seen in the cross-section of the fungal HET-s prion [30]. The 3D reconstructions of the other isolated GPI-anchorless PrP 27–30 fibrils also contained two protofilaments twisting about the fibril axis (S9 Fig). While the general shape and features of protofilaments were remarkably consistent, the four fibrils were structurally heterogeneous with respect to the protofilament orientation (Fig 7), their widths (9.5 nm, 9.4 nm, and 8.7 nm) and crossover distances (76 nm, 67 nm, and 98 nm), which is a common hallmark of amyloid fibrils [31,32], but is also likely to be a reflection of analytical variability, indicating the resolution limitations of our approach. However, the level of resolution attained in these 3D reconstructions allowed us to calculate the average height of a GPI-anchorless PrP 27–30 monomer within each protofilament.

The average protein density has been estimated to be 0.8129 Da/Å^3^ [31,33]. However, considering that a highly compact amyloid might have a slightly different density value, we calculated the density value of a HET-s monomer stacked in a HET-s prion fibril ([30] PDB: EMD-2946). The value obtained was of 0.903 Da/A^3^. Based on a molecular mass of 17,148 Da [18], the calculated molecular volume a GPI-anchorless monomer is 18,990 Å^3^. Consequently, the average height per monomer came to 17.7 Å (Table 1). A similar calculation using the generic 0.8129 Da/Å^3^ protein density value would result in a monomer height of 19.7 Å. These height value calculations confirm that the ~20 Å spacings detected by our two independent single particle analyses originate from individual GPI-PrP^Sc^ subunits stacked along the protofilament axis, lending further support to the 4-rung β-solenoid interpretation for the structure of PrP^Sc^.

**Table 1:** Volumetric analysis of GPI-anchorless PrP 27–30 protofilaments.

	Fibril width (Å)	Protofilament diameter (Å)		Cross-section area (Å^2^)	Height per monomer (Å)
		Max.	Min.		
**Fibril A**	91	50.1	29.3	1,059.25	17.9
**Fibril B**	95	49.0	28.8	1,070.0	17.7
**Fibril C**	94	46.6	35.5	1,177.25	16.1
**Fibril D**	87	43.3	38.5	986.25	19.25
Average (± s.d.)	N/A	N/A	N/A	1,073.2 ± 78.7	17.7 ± 1.3

### Overall structure of the prion fibril

**Fig 8:**
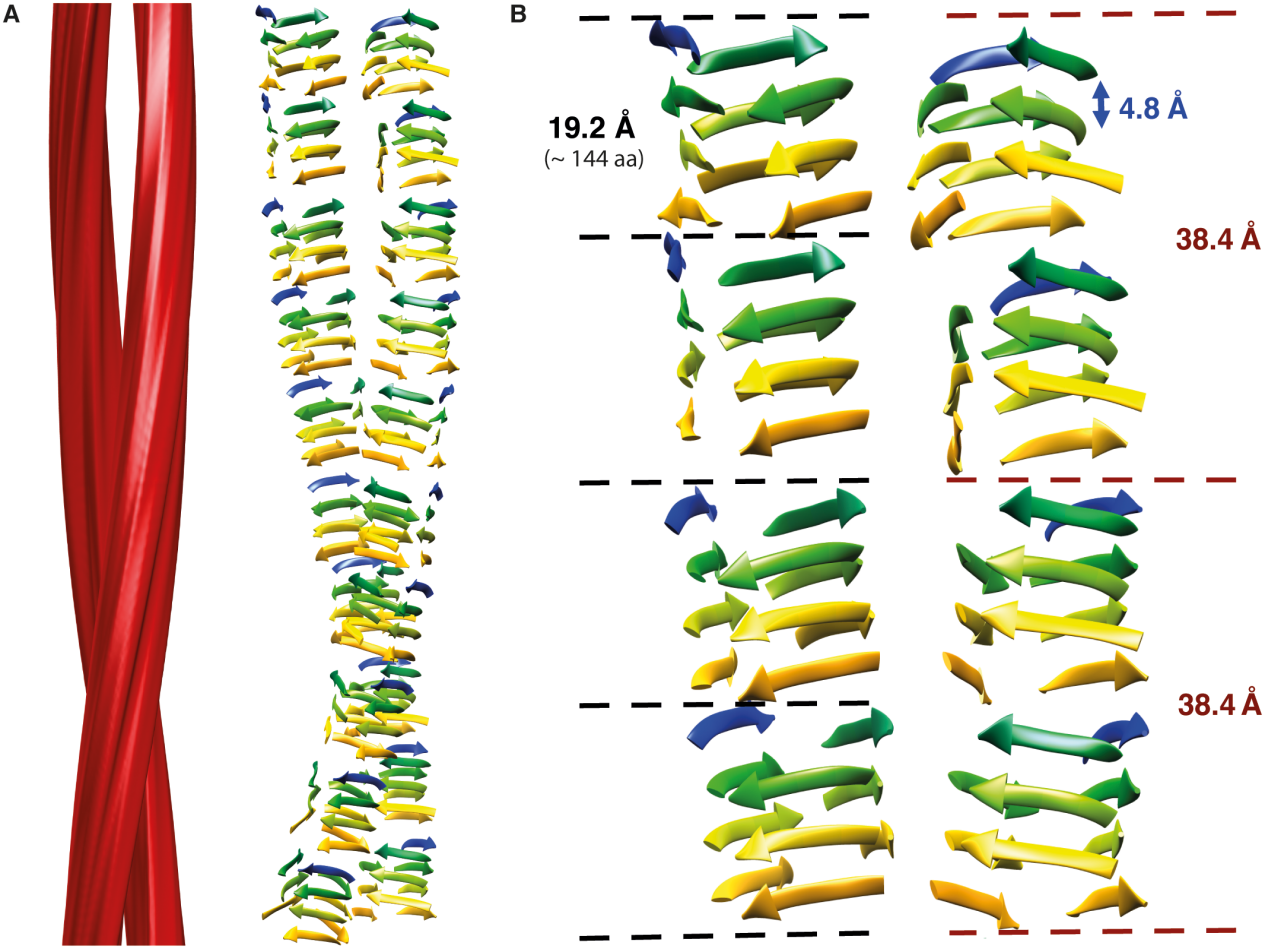
A GPI-anchorless PrP 27–30 fibril and its structural outline. (**A**) 3D reconstruction of an individual GPI-anchorless PrP 27–30 fibril with two protofilaments (left). Cartoon depicting the potential configuration of the polypeptide chains in the PrP 27–30 monomers (right). Please note that this is NOT an atomistic model for the structure of PrP^Sc^. (**B**) Close up view of the possible ß-sheet stacking in a four-rung ß-solenoid structure for illustration purposes only. Different colors represent different ß-solenoid rungs. Characteristic distances of the four-rung ß-solenoid architecture are labeled. One critical constraint for the threading of the PrP sequence into a four-rung solenoid is accommodating the disulfide bridge between cysteines 179 and 214, which is known to be preserved in PrP^Sc^. As in a model of the insulin amyloid, also proposed to be solenoid-like, the two cysteines might be located in register, in corners of two consecutive rungs, with the disulfide bond parallel to the protofilament axis [31,34].

Together, these data support a model in which the structure of PrP^Sc^ and PrP 27–30 consists of a four-rung ß-solenoid with a central, ß-strand-rich core, which is also supported by results obtained with X-ray fiber diffraction [25]. Fig 8 shows a cartoon representing the key elements of the prion architecture surmised from the data obtained in the present studies. It needs to be emphasized that this is not an atomistic model, but a cartoon only—meant to visualize the overall architecture of a four-rung ß-solenoid configuration. In order to account for a molecular height of 19.2 Å (4 x 4.8 Å) the approximately 144 residues of PrP 27–30 must adopt a coiled ß-sheet conformation (Fig 8B). This ~19 Å constraint is particularly relevant because it was obtained across many class averages, i.e. it is not characteristic of just one particular fibril architecture, but rather, emerges as a universal feature of the majority of fibrils present in the preparation. These data agree very well with data obtained from X-ray fiber diffraction of preparations of purified PrP^Sc^ samples of different kinds [25]. Although the actual number of residues in ß-sheet conformation per coil remains unclear, hydrogen-deuterium exchange and limited proteolysis studies have shown that brain-derived PrP^Sc^ consists of several ß-strands connected by short turns and loops [4,18]. Likely, the location of proline residues and the cysteine disulphide bridge pose constraints to the threading of the solenoid, with these elements likely located at corners [31,34].

## Discussion

Our cryo-EM images of GPI-anchorless PrP^Sc^ fibrils, and their subsequent analysis, show that they consist of two intertwined protofilaments, in agreement with a recent study of negatively stained PrP^Sc^ fibrils [35]. Each protofilament exhibits an approximately ellipsoidal cross-section with a linear volume of ~9,900 Å^3^/nm. Given the fact that cryo-EM preserves the native structure of specimens, this information sets a structural restraint for the conformation of GPI-anchorless PrP^Sc^. One important implication is that PrP^Sc^ subunits can only fit into protofilaments with the observed dimensions (Table 1), if they are folded up onto themselves. Based on the routine observation of regular 4.8 Å cross-ß signals in individual GPI-anchorless PrP 27–30 fibrils (Fig 3), a ß-solenoid arrangement is the easiest way to accommodate the peptide into the available protofilament volume.

This arrangement would result in 4 x 4.8 Å (~19 Å) repeats along the protofilament axis, which is exactly what our two independent single particle analyses revealed (Figs 5 and S5), together with an additional ~40 Å signal that likely corresponds to a vertical pairing of two PrP^Sc^ subunits, as seen in HET-s prion fibrils [30]. Therefore, our cryo-EM data revealed a ß-solenoid architecture as the basic element for the structure of the mammalian prion GPI-anchorless PrP^Sc^ (Fig 8), which is in agreement with previous results obtained by X-ray fiber diffraction for other prions such as RML and Sc237 PrP 27–30 [25]. As an important corollary, our cryo-EM data are incompatible with models based on alternative architectures, such as the parallel in-register ß-sheet fold, which is based on a single, superpleated protofilament with a molecular height of only 4.8 Å [6]. It is noteworthy that a β-solenoidal architecture has been demonstrated, as already mentioned, for the HET-s prion [30,36], and has been proposed for insulin [31] and SH3 amyloid [37] fibrils based on cryo-EM data.

While important structural elements still need to be defined, such as which residues participate in the ß-strands that form each solenoid rung, and which ones are located in turns and connecting loops, what we have learned about the structure of GPI-anchorless PrP 27–30 and its four-rung ß-solenoid architecture, allows us to extrapolate about possible templating mechanisms that control the replication of infectious prions *in vivo*. In-phase stacking of identical residues along the peptide chain, as was proposed by the parallel in-register ß-sheet model [6], can be ruled out, as mentioned above, due to the experimentally determined height constraints. Therefore an alternative templating mechanism must explain the replication of prions in general, and the fidelity required for transmitting distinct prion strains in particular [19].

Templating based on a four-rung ß-solenoid architecture must involve the upper- and lowermost ß-solenoid rungs. These edge strands are inherently aggregation-prone, as they are predestined to propagate their hydrogen-bonding pattern into any amyloidogenic peptide they encounter [38]. In fact, the ß-strands of native proteins that contain a ß-solenoid are capped by loops and other structures to block unregulated propagation of ß-sheets. Furthermore, the elimination of the capping structures results in edge-to-edge-driven oligomerization of the "de-capped" ß-solenoids [39]. Thus, it is easy to conceive that these upper and lower ß-solenoid rungs can template an incoming unfolded PrP molecule to create additional ß-solenoid rungs.

It is noteworthy that the molecular forces responsible for the templating − hydrogen-bonding, charge and hydrophobic interactions, aromatic stacking, and steric constraints − are fundamentally similar to those operating during DNA replication. Obviously, the exquisite specificity of the A:T and G:C pairings is lacking and instead, a much more complex array of forces controls the pairing of the pre-existing and nascent ß-rungs. Once an additional ß-rung has formed, it creates a fresh "sticky" edge ready to continue templating until the incoming unfolded PrP molecule has been converted into another copy of the infectious conformer.

Furthermore, the stacking of GPI-anchorless PrP 27–30 molecules into amyloid fibrils, or, in other words, the way in which templating occurs, is either based on a) a head-to-tail orientation resulting in amyloid fibrils with intrinsic polarity (Fig 8), or b) a head-to-head and tail-to-tail orientation, which would result in generally apolar amyloid fibrils. In the former case, templating of ß-sheets would involve direct contact between different parts of the molecule, i.e. heterotypic templating. In the latter case, the same protein stretches will come into contact principally, but homotypic templating would result in two PrP^Sc^ molecules with opposite handedness. Alternatively, a head-to-head arrangement could also rely on heterotypic templating, if different parts of the molecule interact with each other. Experimentally, we observed a ~40 Å signal in Fourier-transform analyses of the fibril segments (Figs 5, S4 and S5) that may originate from a dimeric arrangement. The presence of a distinct dimer signal supports a head-to-head and tail-to-tail arrangement, but, ultimately, higher resolution data are needed to distinguish between these dimer options.

In summary, we present data based on cryo-EM analysis that strongly support the notion that GPI-anchorless PrP^Sc^ fibrils consist of stacks of four-rung ß-solenoids. Two of such protofilaments intertwine to form double fibrils, in agreement with a recent report based on tomography of negatively stained PrP^Sc^ samples [35]. The four-rung ß-solenoid architecture of GPI-anchorless PrP^Sc^ provides unique and novel insights into the molecular mechanism by which mammalian prions replicate.

## Materials and Methods

### Animals

Heterozygous GPI-anchorless PrP transgenic mice (tg44+/-) were developed by Dr. Bruce Chesebro (NIH Rocky Mountain Laboratories, Hamilton, MT, USA) [10]. The mice were crossed to generate homozygous GPI-anchorless PrP animals (tg44+/+) [16] and genotyped by tail DNA analysis using the PCR protocol described in Chesebro *et al*. [10].

### Prion infection of mice

Homozygous GPI-anchorless PrP transgenic mice (tg44+/+) [10,16] were inoculated intra-cerebrally (IC) in the right temporal lobe, at 6 weeks of age, with 20 μl of a 2% RML-infected mouse brain homogenate (BH), prepared in 2X (w/v) PBS. After 365 days post inoculation, the asymptomatic mice were euthanized, the brains were harvested, rinsed in PBS, and stored at -80°C until use.

For the bioassays two groups of six wild-type C57BL/6 mice were inoculated IC with either 20 μl of a 2% GPI-anchorless RML prion BH or with an equivalent amount of purified GPI-anchorless PrP 27–30 resuspended in 5% (w/v) glucose in 2X PBS. Also a new cohort of six wild-type mice was inoculated IC with 20 μl 2X PBS as negative control. Mice were monitored until the appearance of clinical signs, at which time they were euthanized and the brains were removed.

### Ethics statement

Animal experiments were carried out in accordance with the European Union Council Directive 86/609/EEC, and were approved by the University of Santiago de Compostela Ethics Committee (protocol 15005AE/12/FUN 01/PAT 05/JRR2).

### Statistical analysis

The Kaplan-Meier plot survival analyses of the wild-type mouse groups inoculated with GPI-anchorless RML prion BH and purified GPI-anchorless PrP 27–30 were compared using the Gehan-Breslow-Wilcoxon test.

### Immunohistochemistry

Immediately after extraction, the brain was fixed in formalin and then sliced into four transversal sections by cutting the brain caudally and rostrally to the midbrain and at the level of the basal nuclei. The sections were dehydrated by immersion in solutions of progressively higher ethanol concentration and, finally, with xylene before being embedded in paraffin. Haematoxylin-eosin was used to stain 4 μm thick sections. Additional sections were mounted on 3-triethoxysilyl-propylamine-coated glass slides for immunohistochemical (IHC) studies.

IHC for the detection of PrP^Sc^ was performed as follows. Deparaffinized sections were subjected to epitope unmasking treatments: Immersed in formic acid and boiled at low pH (6.15) in a pressure cooker and pre-treated with proteinase K. Endogenous peroxidases were blocked by immersion in a 3% H_2_O_2_ in methanol. Then, the sections were incubated overnight with anti-PrP mAb 2G11 primary antibody (1:100, kindly supplied by Dr. Eoin Monks, University College Dublin, Ireland) and subsequently visualized using the Dako EnVision system K400111/0 (Dako, Glostrup, Denmark) and 3,3’diaminobenzidine as the chromogenic substrate. Additional sections were incubated with a rabbit polyclonal antibody against glial fibrillary acidic protein Z0334, 1:600 (Dako, Glostrup, Denmark) to visualize astrocytic activation. For the glial fibrillary acidic protein detection epitope unmasking treatments were omitted, but the same visualization system was used. As a background control, the incubation with the primary antibodies was omitted.

### Protein purification

GPI-anchorless PrP 27–30 was isolated using a slightly modified version of the method of Baron *et al*. [11]. During the purification, total PrP^Sc^ was treated with 10 μg/ml of proteinase K (PK) at 37°C for 1 h. PK treatment yielded a shortened, protease-resistant form termed PrP 27–30, due to its similarity to GPI-anchored PrP 27–30, which has an apparent molecular mass of 27–30 kDa [40]. The final GPI-anchorless PrP 27–30 pellet was resuspended in 100 μl of deionized water and treated with lipase (porcine pancreas lipase, Sigma No. 62300) at 1 μg/ml for 2 h at 37°C. To trap the fatty acids released by the lipase, bovine serum albumin (BSA) was added to a final concentration of 10 mg/ml. The sample was centrifuged at 22,000 *g* for 20 min, twice, and the pellet containing the PrP 27–30 fibrils was resuspended in 100 μl of deionized water. Finally, fibrils were sonicated with three pulses at a 50% amplitude with a probe ultrasonic homogenizer (Cole Parmer Instrument CO., Chicago, IL, USA), and the purified protein was stored at 4°C or -80°C. The sample purity was assessed by SYPRO Ruby Protein Gel Stain. The yield of GPI-anchorless PrP 27–30 was ~35 μg per mouse brain (BCA protein assay [41]).

### Immunoblotting and SYPRO Ruby protein gel stain

SDS-PAGE was performed in 15% polyacrylamide gels. For Sypro Ruby staining, the gel was washed with ultrapure water, fixed for 1 hour with 10% methanol and 7% acetic acid, followed by overnight incubation with Sypro Ruby solution (Lonza, Rockland, ME, USA) at room temperature with gentle agitation and protection from light. For immunoblotting, the gel was transferred to an Immobilon-P PVDF membrane (Millipore, Billerica, MA, USA) and probed with the 3F10 antibody (recognizing residues 137–151) at a 1:5,000 dilution. Peroxidase-labeled anti-mouse antibody was used as the secondary antibody at a 1:5,000 dilution.

### Electron cryomicroscopy

Samples were prepared by pipetting a 3 μl sample, mixed with a gold fiducial solution (15 nm gold particle size), onto a freshly glow-discharged Lacey carbon grids (Ted Pella Inc., Redding, CA, USA). The grids were plunge-frozen in liquid ethane in a Vitrobot Mark IV (FEI, Eindhoven, The Netherlands). Cryo-EM data were collected using a Titan Krios microscope equipped with Falcon II direct electron detector (FEI, Eindhoven, The Netherlands), operated at 300 kV. Low-dose imaging conditions with 20 electrons per Å^2^ were applied. Images were collected at 1–3 μm underfocus. All micrographs were recorded at a pixel size of 1.34 Å per pixel.

Tilt series data were obtained on a Titan Krios (FEI, Eindhoven, The Netherlands), operated at 300 kV on a Falcon II direct electron detector (FEI, Eindhoven, The Netherlands), under low-dose conditions with 50 electrons per Å^2^. Tomograms of selected areas were obtained from -70 to +70 degrees with a 1.5° tilt increment. The defocus range of the data set was 5–8 μm, with a pixel size of 6 Å. The tilted images were aligned and reconstructed with both the FEI Inspect3D software (FEI, Eindhoven, The Netherlands) and with Tomo3D [42] and the SIRT reconstruction method (30 iterations).

### Measurement of the fibril width

261 fibril widths were obtained from 9 different reconstructed tomograms. Measurements were determined using ImageJ software, following the “Measuring distances between points” manual.

### Image processing and data analysis

Single particle analysis: Only cryo-EM images presenting clear Thon rings were used for processing. The CTF was determined by CTFFIND3 [43] and phases were corrected with Bsoft [44]. Straight fibril segments were extracted manually from 400 micrographs using EMAN’s boxer program [45]. A total of 1305 non-overlapping segments were picked with a box size of 200 x 200 pixels (26.8 x 26.8 nm). In order to align the segments along the y-axis, a symmetrized average from an iterative alignment using the straightest particle in the set as a reference, was used as a starting template. Subsequently, particles were classified by multivariate statistical analysis implemented in IMAGIC [46]. Also, the aligned particles were cut into single protofilaments, realigned and reclassified, resulting in a new data set of 2610 particles. Finally, the summed amplitude spectra of all class averages was calculated and further analyzed.

Reference-free single particle analysis: Image processing was performed with the IMAGIC-4D software [47]. The same 400 selected micrographs (4096 x 4096 pixels) were normalized by *a posteriori* camera correction to remove camera artifacts [48] (S6 Fig). Amplitude spectra were used to determine the CTF parameters and perform CTF-correction by phase flipping (using CTF2D-FIND and CTF2D-FLIP programs). The particles were picked from the best patches of normalized and band-pass filtered micrographs by the PICK-M-ALL program, using a single featureless rectangular reference created by smearing a filament along its length. From the selected patches containing clear fibrils, the best 2725 particles were selected based on standard statistical analyses (averages, standard deviations, cross correlation coefficients). Multivariate eigenvector data compression was then applied, followed by automatic unsupervised classification, to create 20 classes. No alignments were applied to avoid reference bias.

3D fibril reconstruction: Suitable fibril images were selected from a set of 1284 cryo-EM images. An individual fibril, presenting at least half a helical turn (180°), was segmented along the helical axis using EMAN’s boxer program [45] into overlapping boxes of 300 x 300 pixels (40.2 x 40.2 nm). The boxes were centered and spaced 1–5 pixels apart along the helical axis. By measuring the repeat distance of the helical fibril, we could estimate the angular orientation of each box in the set. The underlying assumption was that each box represented a different view of identically the same fibril, where each view is rotated and translated according to the helical twist. By assigning the angles to each box in the set, a preliminary 3D reconstruction was generated by back projection. Two-fold symmetry was then imposed. The preliminary 3D reconstruction was refined against other 3D reconstructions generated from the same fibril using different step sizes [29]. SPIDER software was used for the reconstruction [49]. In order to remove the rippling artifact created by the overlapping segments, the procedure continued with a second stage, using IMAGIC software [46]. The refined 3D reconstruction was rotated 90° around its x-axis and sliced along the yz plane. The new set of cross-sectional projections was aligned, centered, and averaged. The averaged density was symmetrized, replicated along the fibril length, and in-plane rotation angles were assigned based on the helical twist. Finally, the collection of 2D projections was used to assemble the 3D volume. Reconstructions were visualized in UCSF Chimera [50]. The thresholds for the cryo-EM maps were fixed based on the fibril widths measured in the raw images.

The monomer height calculation was accomplished by determining the cross-section area (Å^2^) of each protofilament in Chimera [50] and then calculating the corresponding volume using an experimentally determined molecular mass for the GPI-anchorless PrP 27–30 of 17,148 Da [18], and a mean protein density of 0.903 Da/Å^3^; calculated based on a HET-s monomer stacked in a HET-s prion fibril ([30] PDB: EMD-2946).

## Supporting Information

S1 Fig Representative GPI-anchorless prion fibrils. (**A**) Non-isolated fibrils presenting helical twists or crossovers from the cryo-EM micrographs. (**B**) Isolated straight fibrils selected from cryo-EM images. (**D**) Isolated fibrils, clearly showing a crossover point and suitable for 3D fibril reconstruction. (**D**) Quantitative fibril widths obtained from the tomograms.
(TIF)

S2 Fig Selected reconstructed fibrils extracted from the tomograms. (**A,B**) Fibrils oriented along the Z direction in the tomograms allow us to clearly discern the two protofilaments and the space in-between in the XY slices of the reconstruction. These fibrils are also suitable for 3D visualization, showing their intertwinement. (**C,D**) Fibrils not oriented along the Z direction preclude clear 3D visualization because the effect of the missing wedge smears out the edges of the fibrils. But it is still possible to discern the two protofilaments in some XZ or YZ slices of some reconstructed fibrils as the two shown here. Scale bar: 10 nm. Selected XY, XZ and YZ slices are shown, with the yellow thin lines indicating the correspondence between them. 3D visualizations prepared with Amira (FEI Visualization Sciences Group).
(JPEG)

S3 Fig Single particle picking and 2D classification of GPI-anchorless prion fibril segments. (**A**) Representative cryo-EM micrograph with red boxes indicating fibril segments (particles) that were picked manually. (**B**) Gallery view of the picked particles (left), gallery with the same particles after alignment (right). (**C**) Example of 2D class averages of the fibril segments. Box size is 200 by 200 pixels, equivalent to 26.8 by 26.8 nm. Scale bar, 100 nm.
(TIF)

S4 Fig Averaging of power spectrum over an arc. (**A**). Radial averaging of the logarithm of the summed power spectra (Fig 5A, bottom left) limited to an arc of 20 degrees around the vertical axis (right panel) reveals the signal at 4.8 Å in the plot (left panel). (**B**) Averaging of the Fourier components (Fig 5A, bottom right) along the horizontal axis of the FT, limited to an arc of 40 degrees around the vertical axis (right panel), shows the signals at around 40 Å and 19 Å in the plot (left panel). The Nyquist frequency (2.68 Å) corresponds with the 100 pixel outer border of the spectra.
(TIF)

S5 Fig 2D class averages of individual protofilaments and Fourier-transform analyses. Representative 2D class averages of single protofilaments (**left panel**) and their averaged Fourier-transforms (**middle panel**). The averages are made up of 269, 103 and 120 particles respectively. Single and double arrows indicate 19.1 Å (single pixel) and ~40 Å (38.3 Å and 44.6 Å pixels) signals, respectively. (**Right panel**) Averaging of the Fourier components along the horizontal axis of the FT (see S4 Fig) to better show the signals. The Nyquist frequency (2.68 Å) corresponds with the 100 pixel outer border of the spectra. All box sizes are 200 by 200 pixels, equivalent to 26.8 by 26.8 nm; the rectangular insets (left panel) are 44 by 170 pixels.
(TIF)

S6 Fig *A posteriori* camera correction. **(Left)** Uncorrected raw image patch from the prion dataset showing several vertical and horizontal artifacts. The prion dataset was by coincidence collected on the same camera as used to generate Figs 1 and 2 of Afanasyev et al. [48]. This 512x512 patch represents the worst corner of a 4096x4096 pixel micrograph (collected with a pre-production experimental CMOS camera chip). **(Right)** Corrected image illustrating the successful *a posteriori* normalization procedure.
(TIF)

S7 Fig Flow chart of the 3D reconstruction procedure. **(A)** Segmentation: close up view of an individual fibril, presenting one crossover, segmented in overlapping boxes along its helical axis. **(B)** Assignment of angles: representative particles obtained from the segmentation. Rotation angles were assigned to each one based on the visual helical turn observed in the fibril. **(C)** 3D reconstruction and Refinement: 3D reconstruction obtained in the refinement stage, after the first half of the procedure. Various 3D reconstructions generated from the same fibril, using different overlapping distances (step sizes), were averaged together. Horizontal striations result from the input of the overlapping boxes. **(D)** (**top**) Rotation: cross-sectional projection of the refined 3D map in (C); (**bottom**) Cross-sections: symmetrized average after the centering and alignment of all the 2D cross-sections from the refined 3D reconstruction. This symmetrized average is used to generate **(E)** Final 3D reconstruction. Scale bar, 10 nm.
(TIF)

S8 Fig Cross-sectional projections. **(Top)** 2D cross-sections from the refined 3D reconstructions of the four individual GPI-anchorless prion fibrils analyzed (Fig 4). **(Bottom)** Final symmetrized average cross-sectional projection. This averaged cross-section was used to generate the final 3D maps in S9 Fig. Labels from (**A -D**) are in concordance with Figs 4, 7 and S9.
(TIF)

S9 Fig 3D reconstruction of GPI-anchorless prion fibrils. All panels are arranged identically: (**Left**) Section of a representative electron micrograph showing GPI-anchorless prion fibrils. The single isolated and twisted fibril used for the 3D reconstruction is enclosed by a black box. (**Middle**) Close-up view of the prion fibril. (**Right**) 3D reconstruction of the corresponding GPI-anchorless prion fibril. Note that the contrast of the electron micrographs has been inverted for the subsequent image processing. Scale bars, 100 nm (raw electron micrograph, left panel) and 10 nm (enlarged view and 3D reconstruction, middle and right panels, respectively). Labels from (**A**–**D**) are in concordance with Figs 4 and 7.
(TIF)

S1 Video Electron tomogram from GPI-anchorless prion fibrils. A short movie generated from a 3D tomogram of GPI-anchorless prion fibrils that were embedded in amorphous ice. The tomogram allows the facile visualization of individual fibrils, which would be seen as overlapping structures in a 2D projection. Such tomograms were used for the measurements and reconstructions shown in S2 Fig. The black dots represent fiducial gold that is used to generate the 3D tomogram (15 nm gold particles).
(MOV)

S1 Table Fibril reconstruction statistics from individual GPI-anchorless prion fibrils. Statistics on the four individual GPI-anchorless prion fibrils that were analyzed using a 3D reconstruction approach (Figs 6 and S9). Due to the high degree of lateral association only these four fibrils could be analyzed in this manner.
(DOCX)
