# Supplementary figures and images for: The Structural Architecture of an Infectious Mammalian Prion Using Electron Cryomicroscopy
Source: PLoS Pathog. 2016 Sep 8;12(9):e1005835. doi: 10.1371/journal.ppat.1005835 (PMC5015997; doi:10.1371/journal.ppat.1005835)

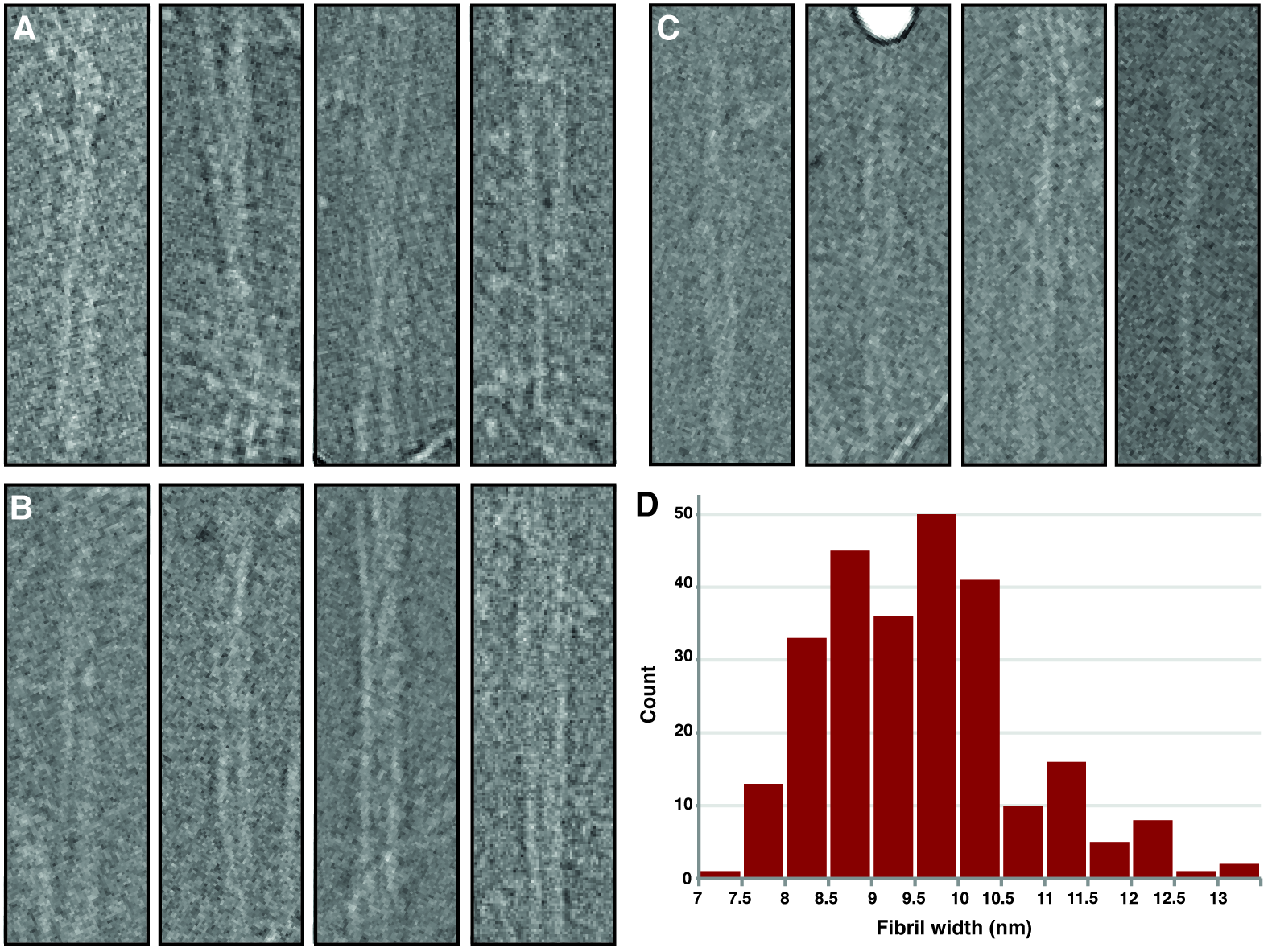

Supplement: S1 Fig — (A) Non-isolated fibrils presenting helical twists or crossovers from the cryo-EM micrographs. (B) Isolated straight fibrils selected from cryo-EM images. (D) Isolated fibrils, clearly showing a crossover point and suitable for 3D fibril reconstruction. (D) Quantitative fibril widths obtained from the tomograms. (TIF) [file ppat.1005835.s001.tif]

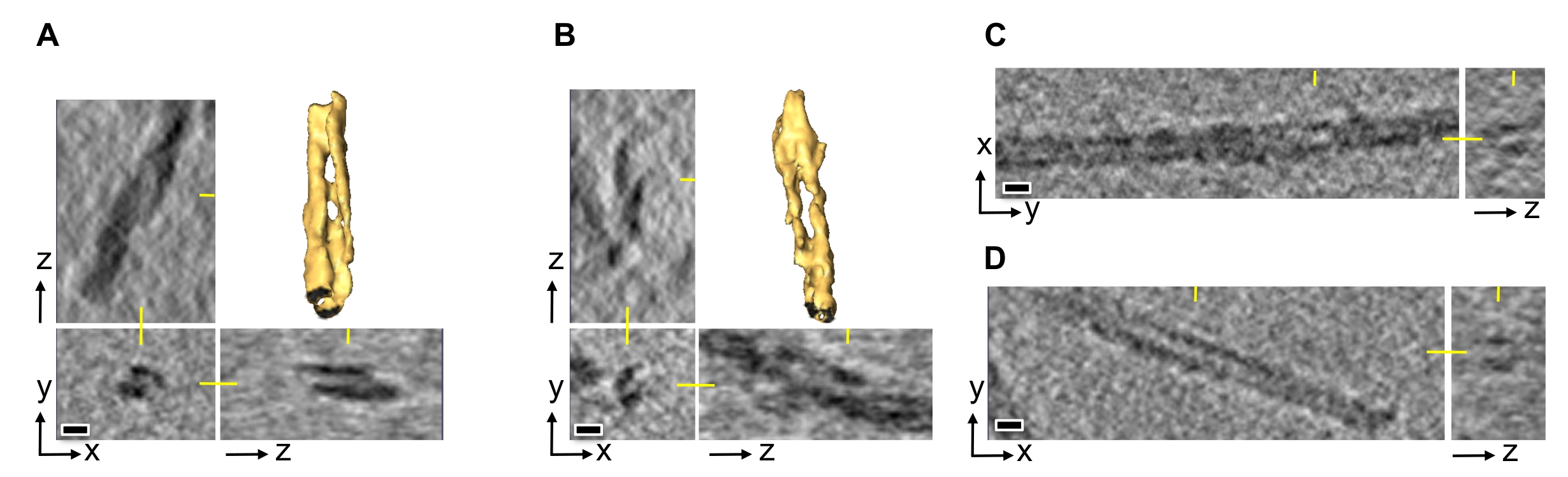

Supplement: S2 Fig — (A,B) Fibrils oriented along the Z direction in the tomograms allow us to clearly discern the two protofilaments and the space in-between in the XY slices of the reconstruction. These fibrils are also suitable for 3D visualization, showing their intertwinement. (C,D) Fibrils not oriented along the Z direction preclude clear 3D visualization because the effect of the missing wedge smears out the edges of the fibrils. But it is still possible to discern the two protofilaments in some XZ or YZ slices of some reconstructed fibrils as the two shown here. Scale bar: 10 nm. Selected XY, XZ and YZ slices are shown, with the yellow thin lines indicating the correspondence between them. 3D visualizations prepared with Amira (FEI Visualization Sciences Group). (JPEG) [file ppat.1005835.s002.jpeg]

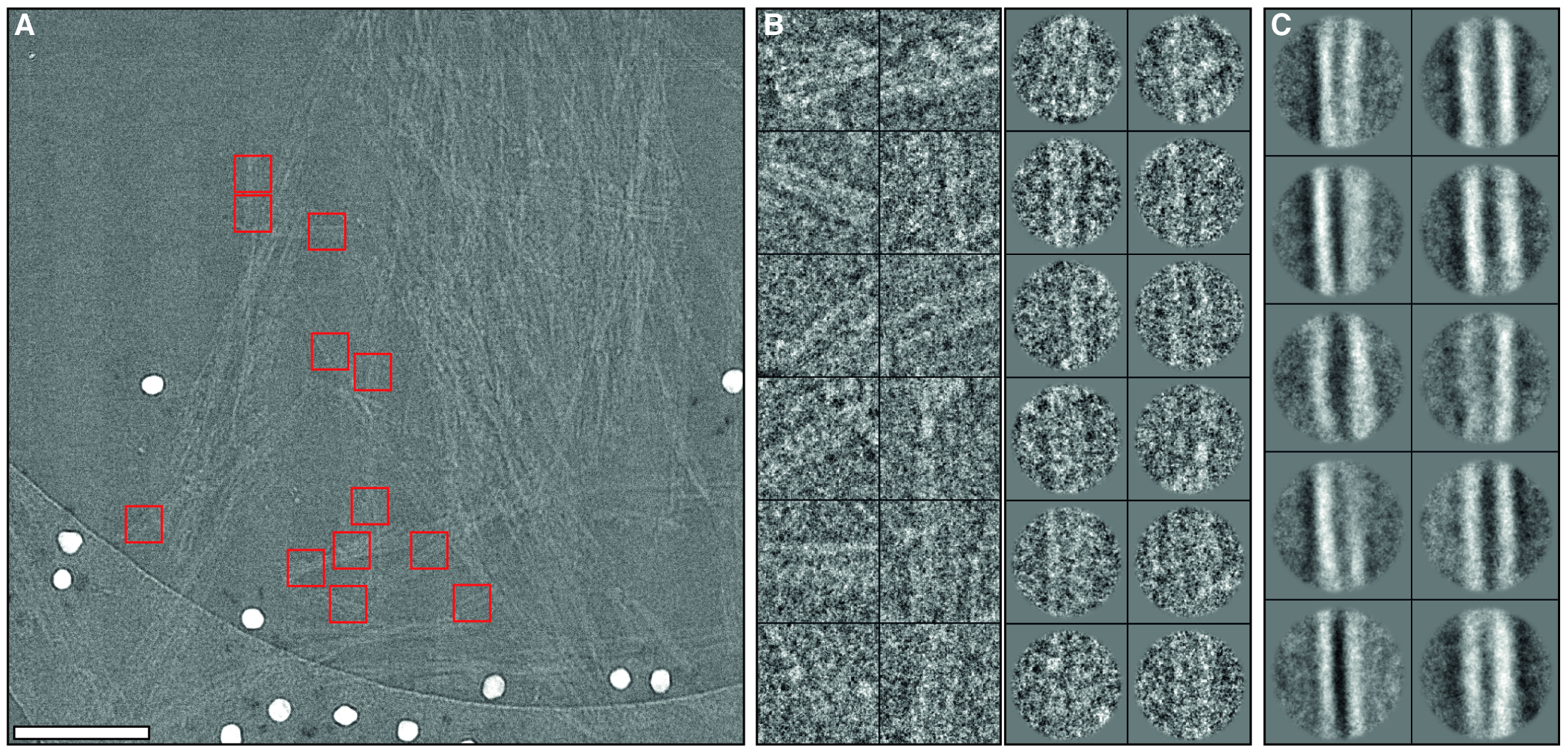

Supplement: S3 Fig — (A) Representative cryo-EM micrograph with red boxes indicating fibril segments (particles) that were picked manually. (B) Gallery view of the picked particles (left), gallery with the same particles after alignment (right). (C) Example of 2D class averages of the fibril segments. Box size is 200 by 200 pixels, equivalent to 26.8 by 26.8 nm. Scale bar, 100 nm. (TIF) [file ppat.1005835.s003.tif]

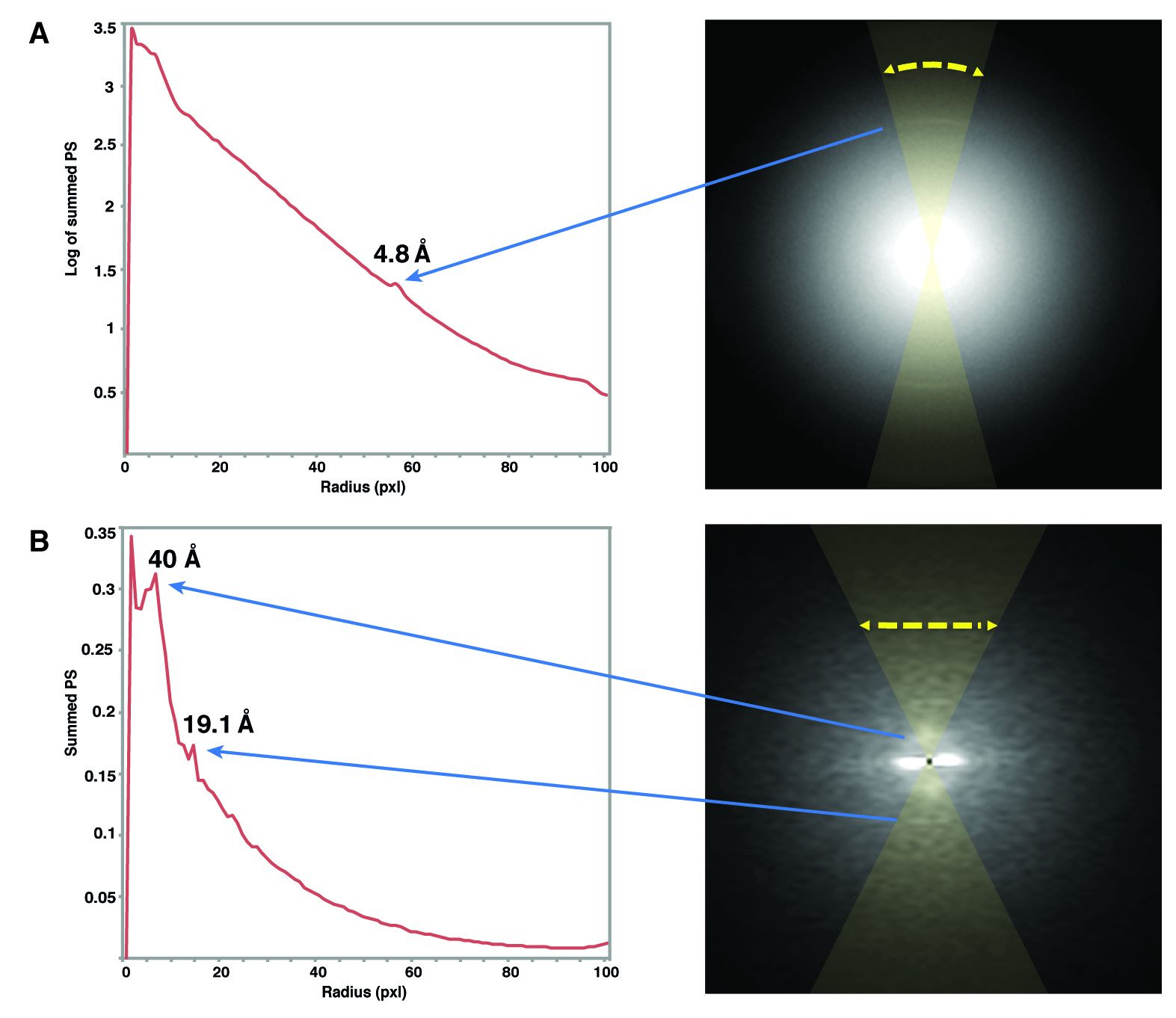

Supplement: S4 Fig — (A). Radial averaging of the logarithm of the summed power spectra (Fig 5A, bottom left) limited to an arc of 20 degrees around the vertical axis (right panel) reveals the signal at 4.8 Å in the plot (left panel). (B) Averaging of the Fourier components (Fig 5A, bottom right) along the horizontal axis of the FT, limited to an arc of 40 degrees around the vertical axis (right panel), shows the signals at around 40 Å and 19 Å in the plot (left panel). The Nyquist frequency (2.68 Å) corresponds with the 100 pixel outer border of the spectra. (TIF) [file ppat.1005835.s004.tif]

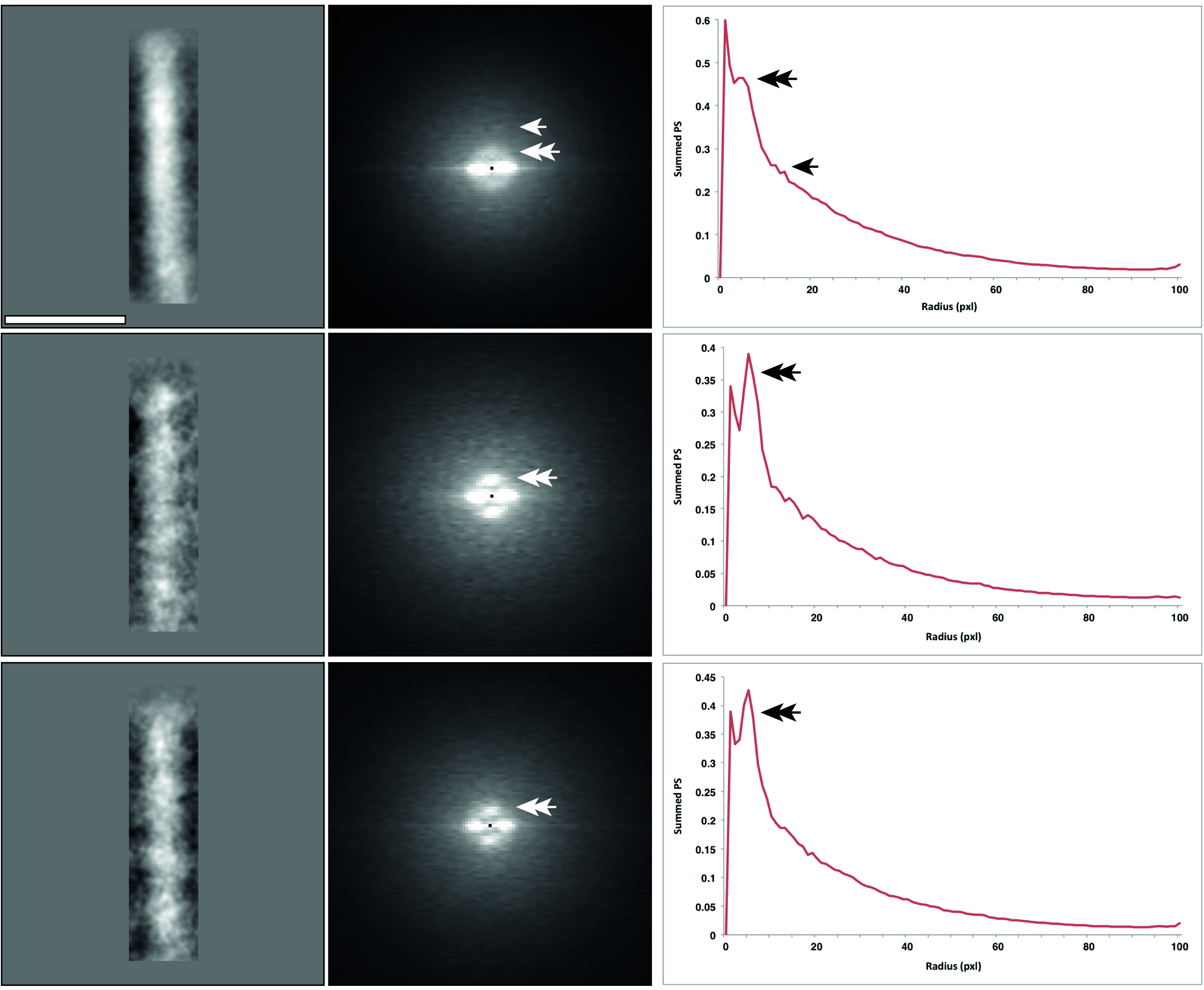

Supplement: S5 Fig — Representative 2D class averages of single protofilaments (left panel) and their averaged Fourier-transforms (middle panel). The averages are made up of 269, 103 and 120 particles respectively. Single and double arrows indicate 19.1 Å (single pixel) and ~40 Å (38.3 Å and 44.6 Å pixels) signals, respectively. (Right panel) Averaging of the Fourier components along the horizontal axis of the FT (see S4 Fig) to better show the signals. The Nyquist frequency (2.68 Å) corresponds with the 100 pixel outer border of the spectra. All box sizes are 200 by 200 pixels, equivalent to 26.8 by 26.8 nm; the rectangular insets (left panel) are 44 by 170 pixels. (TIF) [file ppat.1005835.s005.tif]

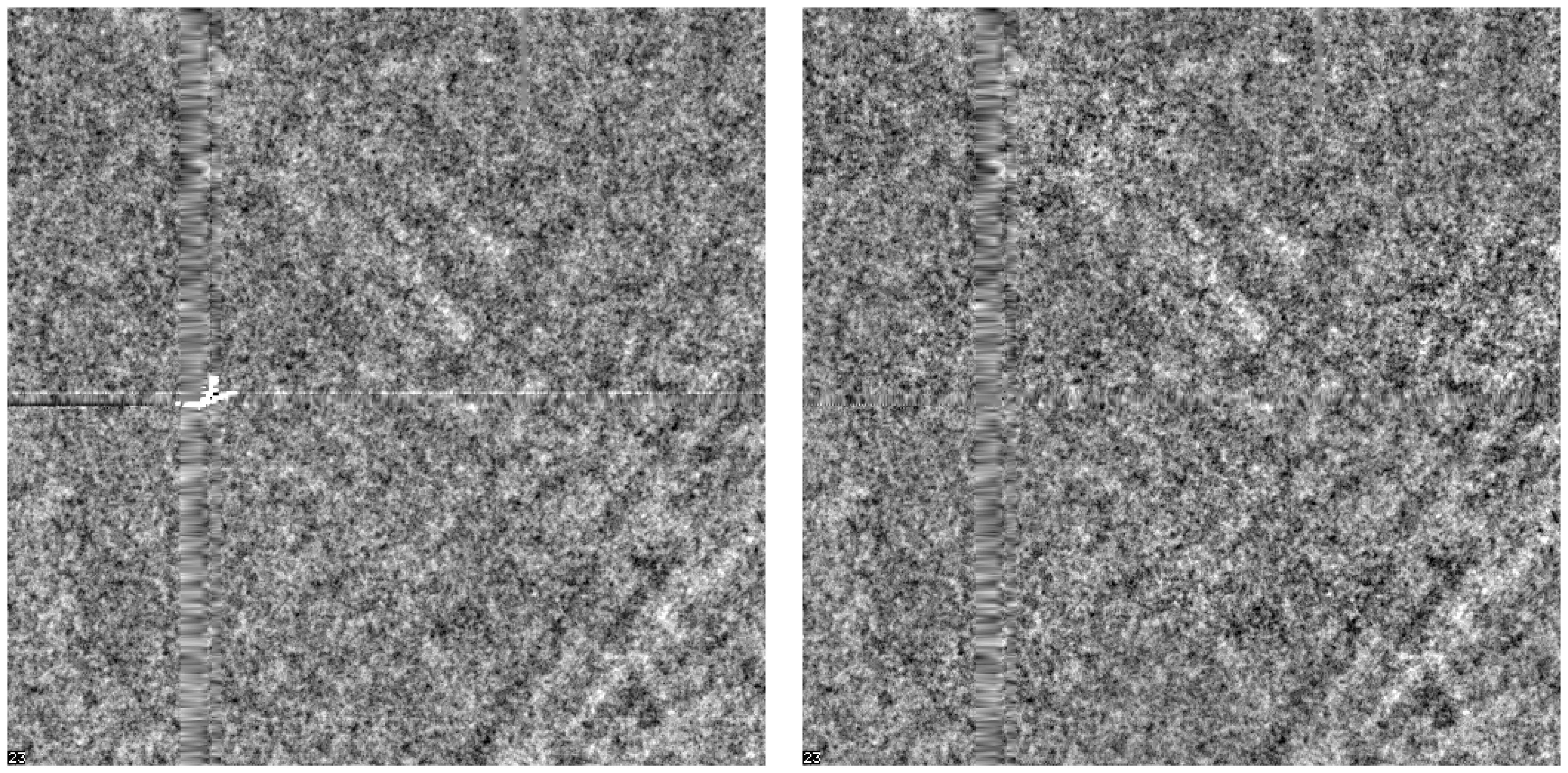

Supplement: S6 Fig — (Left) Uncorrected raw image patch from the prion dataset showing several vertical and horizontal artifacts. The prion dataset was by coincidence collected on the same camera as used to generate Figs 1 and 2 of Afanasyev et al. [48]. This 512x512 patch represents the worst corner of a 4096x4096 pixel micrograph (collected with a pre-production experimental CMOS camera chip). (Right) Corrected image illustrating the successful a posteriori normalization procedure. (TIF) [file ppat.1005835.s006.tif]

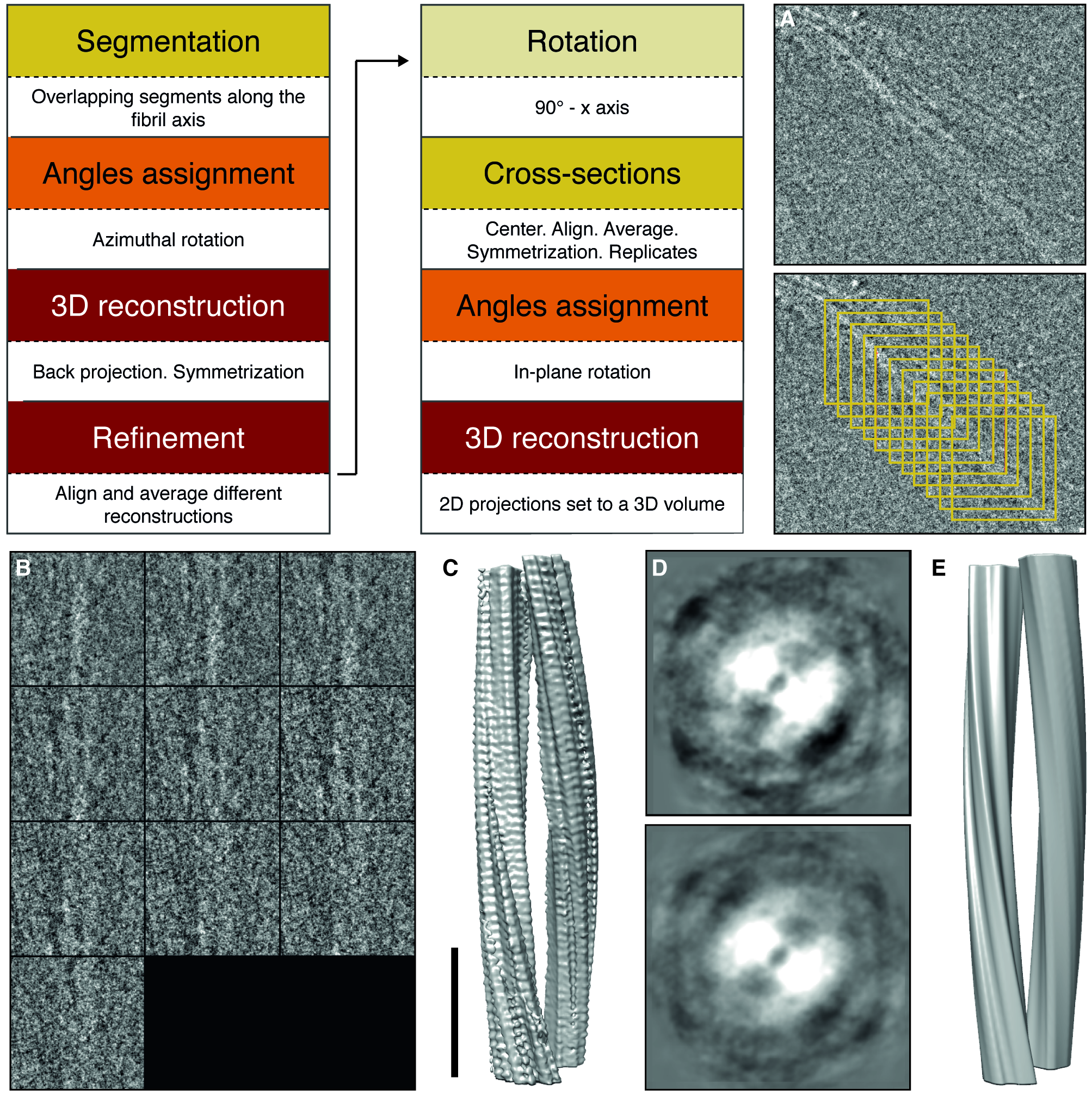

Supplement: S7 Fig — (A) Segmentation: close up view of an individual fibril, presenting one crossover, segmented in overlapping boxes along its helical axis. (B) Assignment of angles: representative particles obtained from the segmentation. Rotation angles were assigned to each one based on the visual helical turn observed in the fibril. (C) 3D reconstruction and Refinement: 3D reconstruction obtained in the refinement stage, after the first half of the procedure. Various 3D reconstructions generated from the same fibril, using different overlapping distances (step sizes), were averaged together. Horizontal striations result from the input of the overlapping boxes. (D) (top) Rotation: cross-sectional projection of the refined 3D map in (C); (bottom) Cross-sections: symmetrized average after the centering and alignment of all the 2D cross-sections from the refined 3D reconstruction. This symmetrized average is used to generate (E) Final 3D reconstruction. Scale bar, 10 nm. (TIF) [file ppat.1005835.s007.tif]

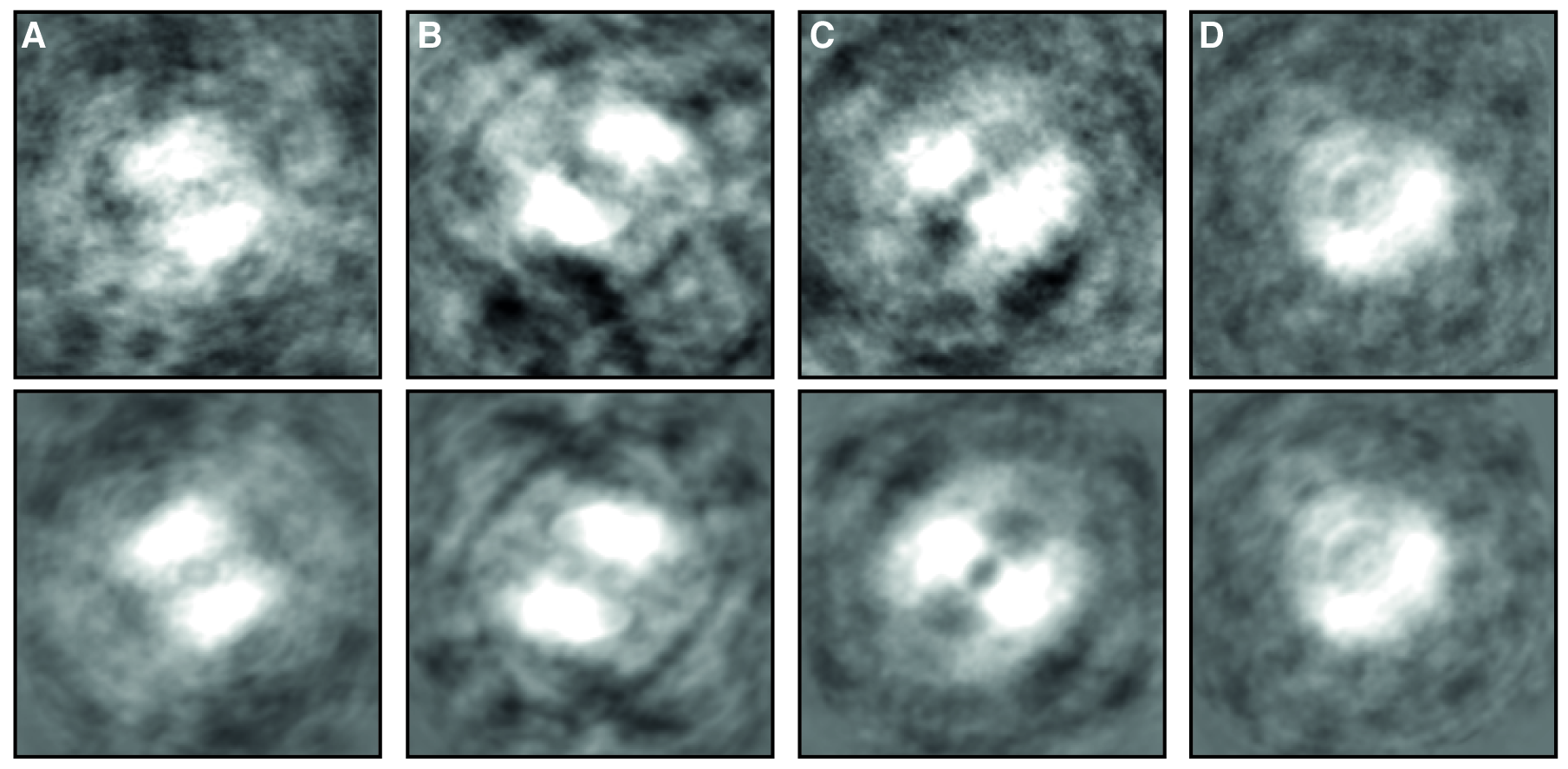

Supplement: S8 Fig — (Top) 2D cross-sections from the refined 3D reconstructions of the four individual GPI-anchorless prion fibrils analyzed (Fig 4). (Bottom) Final symmetrized average cross-sectional projection. This averaged cross-section was used to generate the final 3D maps in S9 Fig. Labels from (A -D) are in concordance with Figs 4, 7 and S9. (TIF) [file ppat.1005835.s008.tif]

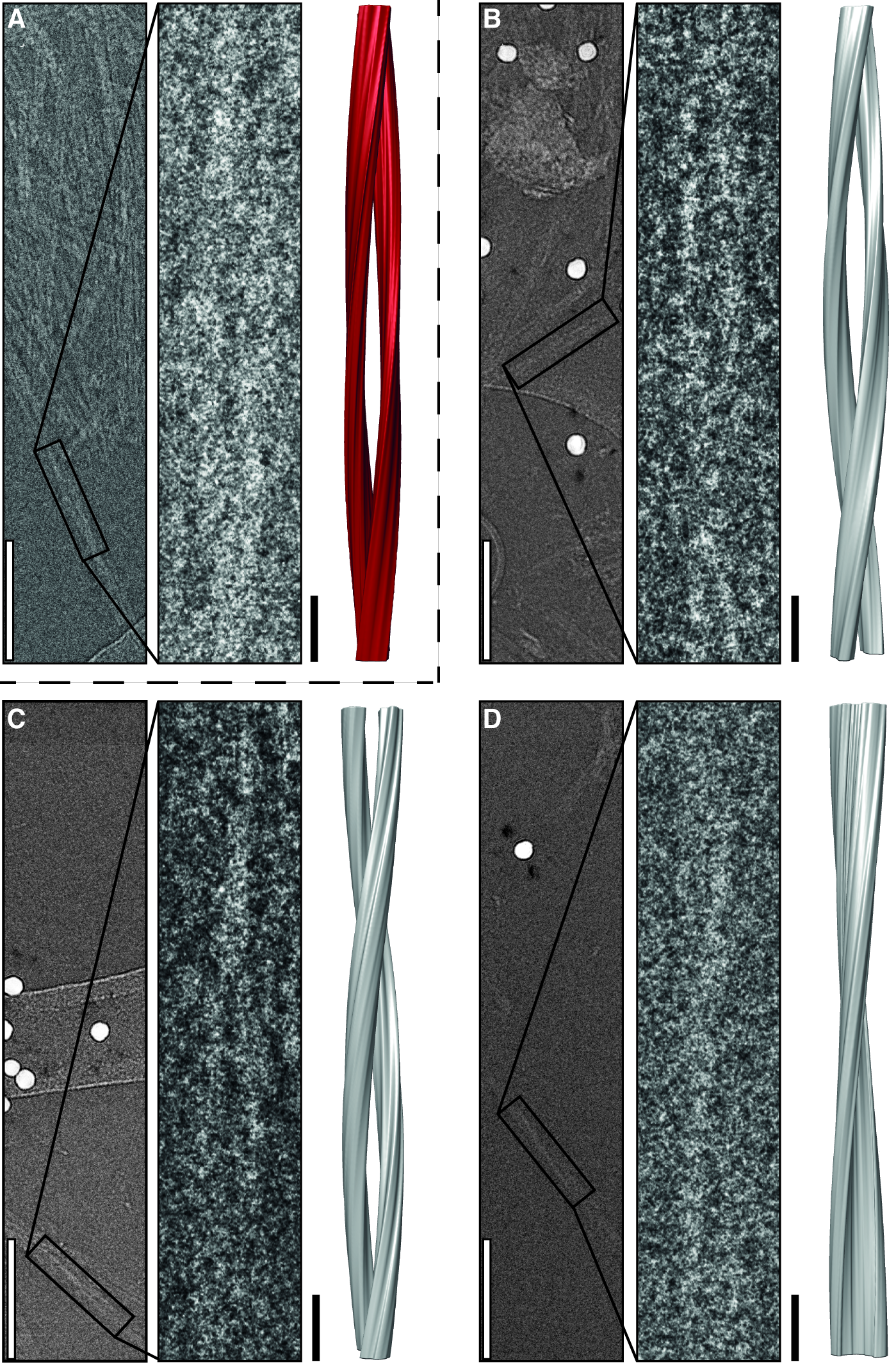

Supplement: S9 Fig — All panels are arranged identically: (Left) Section of a representative electron micrograph showing GPI-anchorless prion fibrils. The single isolated and twisted fibril used for the 3D reconstruction is enclosed by a black box. (Middle) Close-up view of the prion fibril. (Right) 3D reconstruction of the corresponding GPI-anchorless prion fibril. Note that the contrast of the electron micrographs has been inverted for the subsequent image processing. Scale bars, 100 nm (raw electron micrograph, left panel) and 10 nm (enlarged view and 3D reconstruction, middle and right panels, respectively). Labels from (A–D) are in concordance with Figs 4 and 7. (TIF) [file ppat.1005835.s009.tif]
