# Supplementary material for: The Structural Architecture of an Infectious Mammalian Prion Using Electron Cryomicroscopy
Source: PLoS Pathog. 2016 Sep 8;12(9):e1005835. doi: 10.1371/journal.ppat.1005835 (PMC5015997; doi:10.1371/journal.ppat.1005835)
Supplement: S1 Table — Statistics on the four individual GPI-anchorless prion fibrils that were analyzed using a 3D reconstruction approach (Figs 6 and S9). Due to the high degree of lateral association only these four fibrils could be analyzed in this manner. (DOCX) [file ppat.1005835.s011.docx]

**Table S1. Fibril Reconstruction Statistics**

|  | **Fibril** | | | |
| --- | --- | --- | --- | --- |
|  | **A** | **B** | **C** | **D** |
| **Pixel size (Å/pix)** | 1.34 | | | |
| **Box size (pix)** | 300 | | | |
| **Box step size (pix)** | 1, 2, 3, 5 | | | |
| **Total length fibril (Å)** | 632 | 629 | 670 | 854 |
| **Size of 3D reconstruction (Å)** | 402 | | | |
